# The role and application of small extracellular vesicles in gastric cancer

**DOI:** 10.1186/s12943-021-01365-z

**Published:** 2021-04-29

**Authors:** Hao Wu, Mengdi Fu, Jin Liu, Wei Chong, Zhen Fang, Fengying Du, Yang Liu, Liang Shang, Leping Li

**Affiliations:** 1 Department of Gastroenterological Surgery, Shandong Provincial Hospital, Cheeloo College of Medicine, Shandong University, Jinan, 250021 Shandong China; 2 Department of Clinical Medicine, Cheeloo College of Medicine, Shandong University, Jinan, 250021 Shandong China; 3 Department of Gastroenterology, Shandong Provincial Hospital, Cheeloo College of Medicine, Shandong University, Jinan, 250021 Shandong China; 4 Department of Gastroenterological Surgery, Shandong Provincial Hospital Affiliated to Shandong First Medical University, Jinan, 250021 Shandong China; 5 Department of Digestive Tumor Translational Medicine, Engineering Laboratory of Shandong Province, Shandong Provincial Hospital, Jinan, 250021 Shandong China

**Keywords:** Gastric cancer, Small extracellular vesicles, Exosomes, Diagnosis, Therapy, Molecular mechanism

## Abstract

Gastric cancer (GC) is a common tumour that affects humans worldwide, is highly malignant and has a poor prognosis. Small extracellular vesicles (sEVs), especially exosomes, are nanoscale vesicles released by various cells that deliver bioactive molecules to recipient cells, affecting their biological characteristics, changing the tumour microenvironment and producing long-distance effects. In recent years, many studies have clarified the mechanisms by which sEVs function with regard to the initiation, progression, angiogenesis, metastasis and chemoresistance of GC. These molecules can function as mediators of cell-cell communication in the tumour microenvironment and might affect the efficacy of immunotherapy. Due to their unique physiochemical characteristics, sEVs show potential as effective antitumour vaccines as well as drug carriers. In this review, we summarize the roles of sEVs in GC and highlight the clinical application prospects in the future.

## Introduction

As one of the most common malignant tumours, gastric cancer (GC) affects the physical and mental health of those at risk. In a report released by the International Agency for Research on Cancer, the worldwide incidence of GC ranks fifth among all tumours, and mortality ranks fourth [1]. Asia, especially East Asia, has a high incidence of GC. As patients with early GC typically have no symptoms, they often miss the opportunity for optimal treatment. With the advancement of endoscopy technology, an increasing number of GC cases are being diagnosed early, but the invasiveness and high cost of endoscopy restrict large-scale GC screening. At present, there is still a lack of noninvasive and highly accurate tumour biomarkers, which can strong support for the screening of GC, even in the early stages. Despite considerable recent progress in surgical treatment, endoscopic treatment, chemotherapy, radiotherapy, and targeted therapy, the prognosis of patients with advanced GC remains poor, causing a major burden on families and society. Therefore, the application of more indicators of risk and prognosis is urgently needed.

Small extracellular vesicles (sEVs), broadly present nanoscale vesicle structures secreted by almost all cells, can transmit information between cells and participate in their physiological and pathological processes. Due to their unique structural characteristics, sEVs have become hot research topics in recent years, and their role in the initiation and progression of GC is gradually being clarified.

A few years ago, two review articles [2, 3] gave a detailed summary of the role of extracellular vehicles (EVs) or exosomes in GC, which was important and caused an increase in exosome research in the field of GC. Currently, the structure, related technologies and mechanisms of exosomes are being discovered and applied. Most importantly, the International Society for Extracellular Vesicles issued guidelines at the end of 2018 to standardize the nomenclature of extracellular vesicles. Most methods used to isolate exosomes currently co-isolate heterogeneous populations of EVs of diverse biogenic origins. For accuracy and clarity, we refer to “sEVs”, typically called “exosomes” in publications, as small cell-derived membrane vesicles in accordance with the Minimal Information for Studies of Extracellular Vesicles 2018 (MISEV2018) guidelines [4].

This review focused on emphasizing the major advancements in the past few years to reveal the role and application of sEVs in GC. A systematic literature search of Embase, PubMed, Web of Science, and Clinicaltrials.gov was performed for relevant publications up to 1 March 2021. MISEV 2018 guidelines were followed to screen literature about “Stomach Neoplasms”[Mesh], “Exosomes”[Mesh], and “small extracellular vesicles”.

## The biological origin and structural characteristics of EVs

**Fig. 1:**
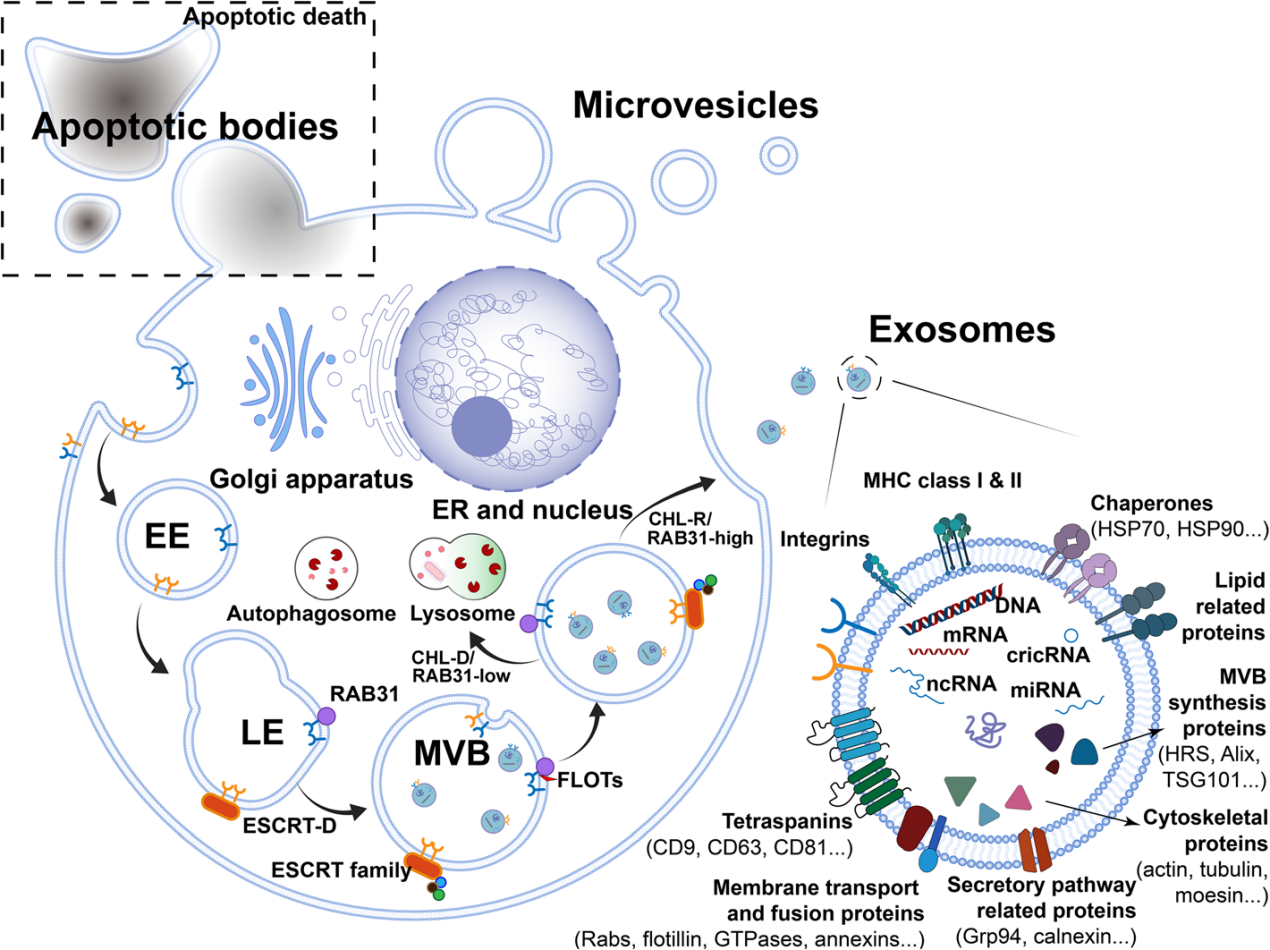
The biogenesis and compositions of exosomes. Golgi apparatus could produce lipid rafts, which facilitate endocytosis. Early endosomes (EE) are formed after the inward budding of the plasma membrane, then they transport from early to late endosomes (LE), which transforming into multivesicular bodies (MVBs) when membrane vesicles sprout inward the cavity. Secretion or degradation is under control of ESCRT family and ESCRT-independent RAB31. Cholesterol-rich (CHL-R) and RAB31-high pre-exosomes are destined to secreted as exosomes, while cholesterol deficient (CHL-D) and RAB31-low are degraded. Exosomes contain complex contents such as proteins, mRNA, miRNA, lncRNA, and DNA. Some protein biomarkers are used to characterize exosomes currently

In 1983, John Stone et al. observed the process by which sheep reticulocytes transform into mature red blood cells and found that red blood cells release transferrin metabolites through some small vesicles, which were initially considered to be cell debris but were later confirmed to be separate structures and named exosomes [5]. Early endosomes are generated after inward budding of the plasma membrane and then transform into multivesicular bodies (MVBs) when membrane vesicles form by budding [6]. MVBs participate in the process of endocytosis and transport of intracellular materials, which involves the sorting, recovery, storage, transportation and release of proteins. MVBs are finally sent to the lysosome for degradation or fuse with the cell membrane and are released into the extracellular environment in a form called exosomes [7]. The most common hypothesis is that the endosomal sorting complex required for transport (ESCRT) family catalyses exosome budding [8]; however, there is no substantive decrease in exosome biogenesis after inhibition of ESCRT family activity [9]. In addition, ESCRT-independent mechanisms, such as heterogeneous nuclear ribonucleoprotein-dependent pathways and neutral sphingomyelinase 2-dependent pathways, were found to affect the formation of exosomes [10]. Recently, Kang et al. [11] identified a novel ESCRT-independent mechanism marked and controlled by RAB31, which has improved our understanding of exosome biogenesis (Fig. 1).

Exosomes are single-cell membrane vesicles with a lipid bilayer membrane structure, a size of approximately 30–150 nm and a density of approximately 1.13–1.21 g/ml [12]. Exosomes are derived from almost all human cells and are widely present in various body fluids, such as blood, urine, cerebrospinal fluid, tears, saliva, milk, ascites, lymph, and amniotic fluid [13].

Plasma membrane-derived ectosomes, also called microparticles or microvesicles, which are 100–1000 nm in diameter, may have largely analogous functions to exosomes [14]. Apoptotic bodies, also called apoptotic vesicles, can be observed when cells undergo apoptosis and may also be confused with other EVs [15].

The function of EVs mainly depends on their rich and complex cargo, of which approximately 76% comprises proteins and 15% mRNAs; the remaining components include DNAs, microRNAs (miRNAs), circular RNAs (circRNAs) and long noncoding RNAs (lncRNAs) [16–20].

However, without optimal separation strategies and specific markers of different sources of EVs at present, it is difficult to propose specific and universal markers of MVB-derived “exosomes” compared with other small EVs. The term “exosomes” was used to refer to EV preparations that have been separated from larger EVs, which refer to a mixture of sEVs of both exosomal and nonexosomal particles [21]. We used “small extracellular vesicles” (diameter < 200 or < 100 nm) in place of “exosomes” according to MISEV2018 [4].

## Separation, characterization and storage of sEVs

A variety of protocols have been applied to the separation of sEVs [22, 23]. Differential ultracentrifugation is currently the most commonly used technique for sEV separation [24]. In addition, other classic techniques, such as density gradients [25], immunoisolation [26], precipitation [27] and filtration [28], are applied. Each method has advantages and disadvantages regarding recovery, specificity, time and cost [29, 30]. Although a number of novel techniques have been developed recently [31–34], absolute isolation of sEVs is still unrealistic, and combinations of methods will continue to be recommended.

Similarly, the identification of sEVs is complicated. Both the source of EVs and the EV preparation must be described quantitatively, according to MISEV2018. Protein content-based EV characteristics are routinely detected and analysed. Positive proteins must include at least one transmembrane/lipid-bound protein (usually CD9, CD63, CD83 and integrin) and one cytosolic protein recovered in EVs (usually ALIX, TSG101, syntenin and HSP70). The levels of at least one negative protein, such as albumin, lipoproteins, and ribosomal proteins, should also be determined. In addition, analysis of functional proteins, such as histones, cytochrome C, calnexin or Grp94, is required when claiming specific analysis of sEVs [4].

Western blotting is the most commonly used method and can detect both surface and internal proteins. Fluorescence microscopes can detect these structures labelled with specific fluorescent probes [35]. Flow cytometry can also be used but is restricted by the small size of sEVs and the low abundance of surface antigens [36]. Mass spectrometry has become economical and accessible in recent years [37]. However, a large number of sEVs are required for protein extraction, which reduces efficiency [6]. At least two different but complementary techniques are also recommended for characterization of the heterogeneity of single vesicles, such as transmission electron microscopy or atomic force microscopy [38].

The storage conditions of sEVs are also very important and may affect their characteristics. There is currently no general consensus that the original samples from which sEVs are extracted should be stored at − 80 °C and used as soon as possible when conducting experiments [39, 40].

## The relationship between sEVs and GC

**Fig. 2:**
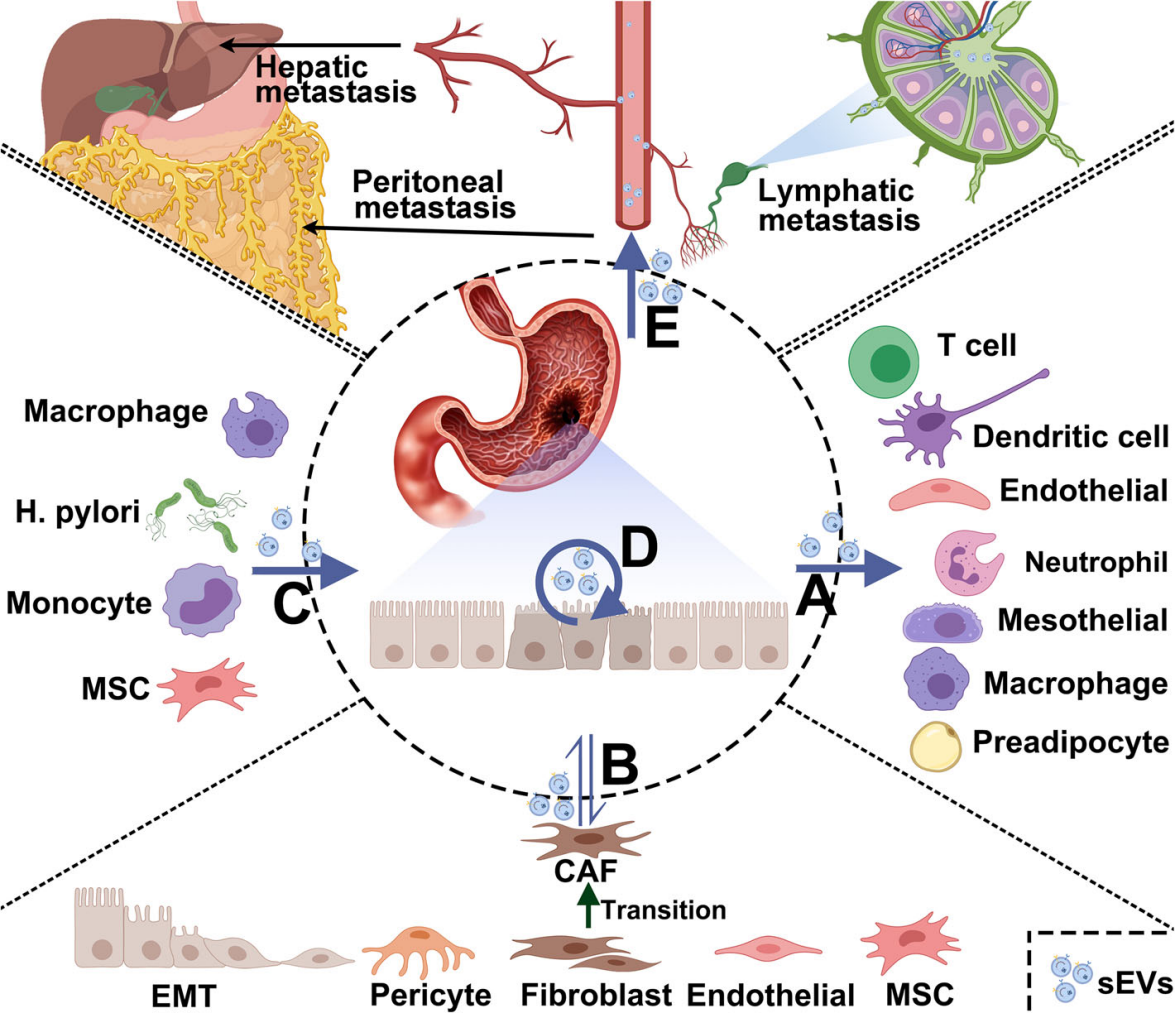
The transmission of sEVs related to GC. **a** GC derived sEVs could promote angiogenesis through endothelial cells and affect different immune cells in tumor microenvironment. They could also differentiate adipocytes into brown-like type. **b** GC derived sEVs could convert pericytes, endothelial, fibroblasts and MSC into CAF, which can transform into tumor microenvironment through different pathway. Also, sEVs secreted by CAF could induce GC progression. **c** Microenvironment-derived and H.pylori-derived sEVs could promote GC progression. **d** GC cells-derived sEVs could promote cancer progression through several contents and signal pathways. **e** GC cells-derived sEVs could promote liver metastasis, peritoneal metastasis and lymphatic metastasis. Abbreviations: GC, gastric cancer; MSC, mesenchymal stem cell; CAF, cancer-associated fibroblast; EMT, epithelial mesenchymal transition; *H. pylori*, Helicobacter pylori

EVs play a relatively important role in the tumorigenesis of GC [41], and their effects on invasion, metastasis, angiogenesis, immune escape, and chemotherapy resistance have been confirmed by several studies (Fig. 2**)**. In 2009, Qu et al. first reported that the sEVs of GC cells can at least partially promote tumour cell proliferation through the PI3K/Akt pathway and MAPK/ERK activation. Metabolism involving the Cbl ubiquitin ligase family and Caspases may also participate in this process [42, 43]. In 2012, Gu et al. confirmed that the TGF-β/Smad pathway mediated by sEVs triggers the differentiation of umbilical cord mesenchymal stem cells into cancer-related fibroblasts. CD97 is also thought to promote the proliferation and invasion of GC cells, and its ability to promote lymphatic metastasis in GC is related to sEVs [44, 45]. In addition, GC cell-derived sEVs can induce infiltration of peritoneal mesothelial cells (PMCs), and infiltrating PMCs in turn promote tumour subserosal invasion [46]. Overall, the interaction between cancer cells and PMCs accelerates gastric wall invasion and peritoneal metastasis. EVs also have an important role in the tumour microenvironment. For example, GC cell-derived sEVs stimulate the phosphorylation of NF-κB in macrophages to promote cancer progression [47] and induce the production of PD-1^+^ tumour-associated macrophages (TAMs), which is beneficial for tumour angiogenesis and metastasis [48]. A similar mechanism of action was reported by Shen et al. [49], and sEVs promote the polarization of N2 tumour-associated neutrophils to induce autophagy and increase tumour activation, promoting GC migration [50]. Ji et al. [51] and Wang et al. [52] revealed the role of sEVs in 5-Fluorouracil (5-FU) and platinum resistance. These studies contribute to the discovery and application of potential alternative drugs to reverse resistance.

Although the study of sEVs related to GC is still in early stages, the quantity and quality of related research have improved recently, providing new insight into the mechanisms underlying the initiation and progression of GC. In the following sections, we discuss the interaction between GC and sEV cargo in the tumour microenvironment and in GC cells to provide a comprehensive understanding of the relationship between them.

### sEVs and GC initiation & progression

The initiation and progression of cancer are often affected by the mutual influence of tumour cells or the microenvironment, but the mechanism is not completely clear. Under physiological and pathological conditions, sEVs are released from the cell membrane, and the biologically active substances carried as cargo are involved in many processes (Table 1).

**Table 1:** Role of sEVs cargo in initiation and progression of GC

Type	Contents	Donor cells	Recipient cells	Function	Ref.
protein	CD97	SGC-7901	SGC-7901	Promote cell proliferation and invasion	[45]
protein	FZD10	HGC-27 and N-87	HGC-27 and N-87	Sustain cancer cell proliferation	[53]
protein	UBR2	p53^−/−^mBMMSC	p53^+/+^ mBMMSC and MFC	Promote cell proliferation, migration, and stemness	[54]
protein	GKN1	HFE-145	AGS and MKN1	Inhibit gastric tumorigenesis	[55]
protein	TRIM3	Overexpressed MGC-803 and SGC-7901	MGC-803 and SGC-7901	Suppress GC growth	[56]
miRNA	miR-15b-3p	SGC-7901 and BGC-823	SGC-7901 and BGC-823	Enhance tumorigenesis and malignant transformation	[57]
miRNA	miR-221	GC-MSCs	HGC-27	Promote cell proliferation and migration	[58]
miRNA	miR-221	BM-MSCs	BGC-823 and SGC-7901	Enhance cell proliferation, migration, invasion, and adhesion to the matrix	[59]
miRNA	miR-1290	SGC-7901 and AGS	SGC-7901 and AGS	Promote proliferation and invasion	[60]
miRNA	miR-423-5p	SGC-7901 and HGC-27	SGC-7901 and HGC-27	Promote cancer growth	[61]
miRNA	miR-155-5p	GES-1 and AGS	AGS	Promote proliferation and migration	[62]
miRNA	miR-301a-3p	Hypoxia MGC803	MGC803 and MKN45	Promote GC progression	[63]
lncRNA	SPRY4-IT1	BGC-823	MKN-28	Promote cell growth	[64]
lncRNA	ZFAS1	BGC-823 and MGC-803	MKN-28	Enhance cell proliferation and migration	[65]
lncRNA	lncHEIH	HGC-27	GES-1	Promote GC progression	[66]
lncRNA	lnc01559	MSCs	HGC-27 and AGS	Promote GC progression	[67]
circRNA	circNRIP1	BGC-823 and MKN-45	BGC-823 and MKN-45	Promote GC progression	[68]
circRNA	circSHKBP1	BGC-823 and HGC-27	BGC-823 and HCG-27	Promote GC progression	[69]
circRNA	circNHSL1	HGC-27 and AGS	HGC-27 and AGS	Contributes to GC progression	[70]

*Abbreviations*: *mBMMSC* mouse bone marrow mesenchymal stem cell, *MSC* mesenchymal stem cell, *HUVECs* human umbilical vein endothelial cells, *TAM* tumor-associated macrophage

Although many studies have explored the relationship between sEVs and early gastric cancer, only a few articles have clarified the mechanism of sEVs in gastric carcinogenesis. Yoon et al. [55] identified Gastrokine 1 (GKN1) through a protein microarray in 2018 and found that it binds to 27 sEV proteins. GKN1 in sEVs can inhibit the proliferation of a variety of GC cells and induce apoptosis, and the results were supported in vitro. After further research, GKN1 was confirmed to be a tumour suppressor that reduces the initiation of GC by decreasing activation of the Hras/Raf/MEK/ERK signalling pathway, but its specificity is high, and the same effect has not been found in other gastrointestinal tumours [71]. Wei et al. [57] used qRT-PCR to show that miR-15b-3p is highly expressed in tissues, serum, cells and sEVs, enhancing the tumorigenesis and malignant transformation of GC by inhibiting the NYDLT1/Caspase-3/Caspase-9 pathway and suppressing apoptosis in GC. Based on these findings, it was proposed that miR-15b-3p can serve as a diagnostic and prognostic biomarker for GC. In addition, more articles have elucidated the mechanism of tumorigenesis through the exploration of the microenvironment, which we will describe later in the article.

Regarding the malignant behaviours of tumour cells, such as proliferation and invasion, many molecules have also been discovered. The FZD protein family of receptors in the Wnt signalling pathway plays an important role in gastrointestinal tumours. In cells with silencing of Frizzled 10 (FZD10) expression, addition of FZD10 and FZD10 mRNA restored viability. FZD10 has been shown to be a potential messenger of cancer reactivation and may play an equally active role in distant metastasis [53]. Mesenchymal stem cells (MSCs) are present in the tumour microenvironment. The N-recognin 2 (UBR2) component of ubiquitin protein ligase E3 can be delivered to target cells by sEVs to promote the growth and metastasis of GC [54]. Both GC tissue-induced mesenchymal stem cells [58] and bone marrow-induced mesenchymal stem cells [59] have been reported to significantly promote the growth and migration of GC cells through paracrine miR-221, the expression levels of which are closely related to lymphatic metastasis, venous infiltration and TNM staging. For instance, low expression of lncRNA SPRY4-IT1 caused dividing GC cells to stagnate in G1 phase, inhibiting the proliferation of GC cells. Overexpression of lncRNA SPRY4-IT1 promoted cell migration and invasion through the SPRY4-IT1/miR-101-3p/AMPK axis. SPRY4-IT1 has been shown to be transported in sEVs and is related to the progression and metastasis of GC [64].

Zhang et al. [68] detected an increased level of circNRIP1 expression in GC through RNA-seq analysis and showed that it has a tumour-promoting effect. Subsequently, the circNRIP1-miR-149-5p-AKT1/mTOR axis was found to be an important mechanism that promotes the proliferation, migration and invasion of GC, as determined by Western blot analysis and immunofluorescence. In addition, this molecule enhanced the stability of VEGF mRNA, as proven by in vivo and in vitro experiments. Moreover, circSHKBP1 can directly bind to HSP90 and hinder its interaction with STUB1, inhibiting the ubiquitination of HSP90 and promoting the proliferation, migration, invasion and angiogenesis of GC cells [69]. Recently, Zhang et al. showed that degradation of miR-30a or miR-30b could be induced by HOTAIR to promote the carcinogenesis of GC [72]. In addition, other cargo [60–62, 65–67, 70] plays a role in tumorigenesis and tumour progression.

Tumour cells develop significantly faster than vascular cells due to their strong proliferation, so hypoxia is often observed, and oxygen-monitoring mechanisms might be activated [73]. Xia et al. [63] found that hypoxia GC-derived sEV-enriched miR-301a-3p could target PHD3 to inhibit HIF-1α degradation. The sEV-miR-301a-3p/HIF-1α signalling axis facilitated GC proliferation and even metastasis.

Other studies have revealed that sEVs promote tumour progression through mechanisms such as the NF-κB pathway [47], Hedgehog pathway [74], and PI3K-Akt pathway [75]. Nonetheless, the contents of sEVs that play critical roles need to be further explored.

### sEVs and angiogenesis & metastasis

Inhibiting gastric cancer-related angiogenesis is a current targeted treatment strategy for GC, and many studies involving coculture of GC cell sEVs with human umbilical vein endothelial cells (HUVESs) have been performed (Table 2). Studies have found that miR-130a [79], miR-135b [80], miR-155 [81, 82], miR-23a [83], X26nt [89] and YB-1 [76] promote angiogenesis through different mechanisms and proposed potential targets beyond conventional targeted drug therapy. A recently published article claimed that sEVs containing miR-6785-5p could suppress angiogenesis and metastasis in GC by inhibiting INHBA [84].

**Table 2:** Role of sEVs cargo in angiogenesis and metastasis of GC

Type	Contents	Donor cells	Recipient cells	Function	Ref.
protein	TRIM3	Overexpressed MGC-803 and SGC-7901	MGC-803 and SGC-7901	Suppress GC metastasis	[56]
protein	YB-1	SGC-7901	HUVECs	Promote angiogenesis	[76]
protein	EGFR	SGC-7901	Liver cells	Promote GC liver metastasis	[77]
protein	Wnt5a	LNM-derived GC cells	BM-MSCs	maintaining tumour-promoting phenotype and function	[78]
miRNA	miR-130a	SGC-7901	HUVECs	Promote angiogenesis and tumour growth	[79]
miRNA	miR-135b	SGC-7901	HUVECs	Promote angiogenesis	[80]
miRNA	miR-155	SGC-7901	HUVECs	Promote growth, metastasis, and tube formation of vascular cells	[81, 82]
miRNA	miR-23a	HGC-27	HUVECs	Promote angiogenesis	[83]
miRNA	miR-6785-5p	HUVECs	MGC-803 and SGC-7901	Suppress angiogenesis and metastasis	[84]
miRNA	miR-196a-1	GSU and N-87	GSU and N-87	Promote GC cell invasion and metastasis	[85]
miRNA	GKN1	HFE-145	AGS and MKN1	Suppress migration and invasion of GC cells by inhibiting epithelial-mesenchymal transition.	[71]
miRNA	miR-29	Peritoneal fluid	/	Suppress growth of disseminated peritoneal tumour cells	[86]
miRNA	miR-21-5p	MGC-803, MKN-45, HGC- 27, and SGC-7901	PMC and HMrSV5	Induce MMT and promote tumour peritoneal metastasis	[87]
miRNA	miR-423-5p	Overexpressed SGC-7901 and HGC-27	SGC-7901 and HGC-27	Promote GC metastasis	[61]
miRNA	miR-301a-3p	Hypoxia MGC803	MGC803 and MKN45	Promote GC metastasis	[63]
lncRNA	PCGEM1	HGC	NGC	Promote invasive and metastasis	[88]
ncRNA	X26nt	MGC-803	HUVECs	Increases angiogenesis and vascular permeability	[89]
circRNA	circ-RanGAP1	HGC-27 and AGS	HGC-27 and AGS	Promote GC invasion and metastasis	[90]

The premetastatic niche, a preformed microenvironment influenced by sEVs secreted from the primary site to distal metastasis, has been reviewed in many kinds of tumours [91–93]. Substantial in vitro and preclinical models have proven that sEV-driven transfer of biomolecular cargo between tumour and normal cells potently promotes distal microenvironments that are favourable to cancer growth [94]. A previous study demonstrated that integrin αvβ5 in sEVs was associated with liver metastasis [95]. In addition, sEVs containing secretory EGFR derived from GC cells effectively activate hepatocyte growth factor, which in turn binds to c-MET receptors on migrating cancer cells to promote the homing of metastatic cancer cells. Therefore, EGFR may be beneficial to the development of a liver-like microenvironment, leading to liver-specific metastasis [77]. Moreover, miR-196a-1 could promote metastasis to the liver in vitro and in vivo via sEVs from highly invasive to low-invasive GC cells [85]. Unfortunately, no more sEV-driven molecules that can indicate organ-specific metastasis have been discovered. We will describe the the relationship between sEVs and the microenvironment later in the article.

Peritoneal metastasis is a manifestation of advanced GC and often indicates a poor prognosis, and mesothelial cells function in the protective barrier of the peritoneum. EVs derived from GC can destroy the mesothelial barrier and cause peritoneal fibrosis, promoting peritoneal metastasis [96]. Similarly, miR-21-5p induced the mesothelial-to-mesenchymal transition (MMT) in peritoneal mesothelial cells and promoted peritoneal metastasis [87]. Using peritoneal lavage or ascites of patients with GC, Ohzawa et al. observed differences in the expression of miR-29 in patients with or without peritoneal metastasis; patients with low expression of miR-29b-3p had a higher peritoneal metastasis rate and a worse prognosis. MiR-29 may play an inhibitory role in the growth of diffuse peritoneal tumour cells [86].

Lymphatic metastasis is a common metastatic route of GC. Lu et al. studied the miR-877-3p/VEGFA axis mediated by circ-RanGAP1 and found that high expression of circ-RanGAP1 is closely related to TNM staging, lymphatic metastasis and poor survival [90]. A recently published study showed that bone marrow-derived mesenchymal stem cells could be specifically educated by sEVs containing Wnt5a via activation of the YAP signalling pathway [78]. This new insight into the mechanisms of lymphatic metastasis suggests potential therapeutic targets.

In addition, Piao et al. conducted a very interesting experiment in which they cocultured GC cells in conditioned medium from GC cells grown under hypoxia or normoxia and found that lncRNA PCGEM1 was highly expressed in GC cells cultured under hypoxic conditions and induced the invasion and metastasis of GC cells under normoxic conditions. This finding may be because PCGEM1 reduces the degradation of SNAI1, which in turn induces the epithelial-mesenchymal transition in GC; this finding may explain the molecular mechanism of GC cell invasion under hypoxic conditions [88]. Fu et al. discovered TRIM3 by screening the proteomic profile of serum sEVs in patients with GC and reported that it inhibits the growth and metastasis of GC in vitro and in vivo by regulating stem cell factors and EMT regulators [56].

### sEVs and chemoresistance

Advanced unresectable GC usually involves comprehensive treatment based on drug therapy, commonly including chemotherapy and targeted therapy. However, in clinical practice, the same chemotherapy regimen often produces different therapeutic effects in different patients; some patients even develop chemotherapy resistance early, which is associated with poor prognosis and poses considerable therapeutic challenges. Many previous studies on the analysis of disease-related genes, combined with the molecular classification of GC, have been carried out to provide comprehensive treatment guidance for patients. With the development of sEV research in recent years, whether the contents of sEVs have an impact on chemotherapeutic resistance mechanisms and even the immune microenvironment has naturally become an emerging hot research topic [51]. sEVs might also have broad prospects for surmounting multidrug resistance in cancer [97] (Table 3).

**Table 3:** Role of sEVs cargo in chemoresistance of GC

Drug	Contents	Type	Function	Ref.
Cisplatin	RPS3	protein	Promote the chemoresistance of cisplatin-sensitive cells	[98]
	miR-21	miRNA	Tumour-associated macrophages derived sEVs-miR-21 confer cisplatin resistance in GC	[99]
	anti-miR-214	miRNA	Reverse the resistance to cisplatin in GC	[52]
	miR-522	miRNA	Inhibit ferroptosis in cancer cells by targeting ALOX15 and blocking lipid-ROS accumulation	[100]
	miR-500a-3p	miRNA	Could be a potential modality for the prediction and treatment of GC with chemoresistance	[101]
	HOTTIP	lncRNA	Contribute to cisplatin resistance in GC cells by regulating miR-218/HMGA1 axis	[102]
	circ-0000260	circRNA	Contribute to cisplatin resistance by upregulating MMP11 via targeting miR-129-5p.	[103]
	circ-PVT1	circRNA	Contribute to cisplatin resistance via miR-30a-5p/YAP1 axis.	[104]
Oxaliplatin	circ-0032821	circRNA	Contribute to oxaliplatin resistance by regulating SOX9 via miR-515-5p	[105]
5-FU	TFAP2E	protein	Hypermethylation of TFAP2E resulted in 5-FU chemoresistance in GC cells	[106]
Doxorubicin	miR-501	miRNA	Resistance to doxorubicin is possibly achieved by sEVs-miR-501-induced downregulation of BLID, subsequent inactivation of caspase-9/− 3 and phosphorylation of Akt.	[107]
	miR-223	miRNA	Promote doxorubicin resistance in GC cells by inhibiting FBXW7.	[108]
Vincristine	CLIC1	protein	Induce the development of resistance to vincristine in vitro, which might relate to up-regulated P-gp and Bcl-2.	[109]
Paclitaxel	miR-155-5p	miRNA	Induce EMT and chemo-resistant phenotypes from paclitaxel-resistant GC cells to the sensitive cells, which may be mediated by GATA3 and TP53INP1 suppression.	[110]

Platinum drugs are one of the most common chemotherapy drugs used for GC, and cisplatin and oxaliplatin have been included as first-line treatments. Cisplatin-resistant gastric cancer cells communicate with sensitive cells through RPS3 in sEVs and activation of the PI3K-Akt-cofilin-1 signalling pathway [98]. Zheng et al. [99] found that M2-polarized macrophages can promote resistance to cisplatin in GC cells. sEV-miR-21 can be directly transferred from macrophages to GC cells, downregulating PTEN expressing and activating the PI3K/AKT signalling pathway to inhibit apoptosis. Moreover, a newly discovered form of iron-dependent oxidative cell death called ferroptosis is caused by lethal accumulation of lipid-based reactive oxygen species (ROS) [111], which may be related to tumour progression and drug resistance. Zhang et al. [100] found that arachidonic acid lipoxygenase 15 (ALOX15) is closely related to the production of lipid-based ROS in GC. The most valuable finding is that sEVs-miR-522 secreted by tumour-associated fibroblasts is a potential inhibitor of ALOX15, blocking the accumulation of lipid-based ROS and inhibiting ferroptosis in GC cells and thereby reducing sensitivity to chemotherapy drugs such as cisplatin and paclitaxel. Furthermore, miR-500a-3p and miR-214 are reportedly related to platinum resistance, and anti-miR-214 can reverse the resistance of GC to cisplatin. Perhaps in the near future, these molecules will become adjuvant drugs for the treatment of cisplatin-resistant GC [101–104]. A similar mechanism has also been reported for oxaliplatin resistance [105].

The drug 5-fluorouracil (5-FU) is another commonly used chemotherapy agent for GC. It has been reported [106] that hypermethylation of transcription factor activation promoter-binding protein 2e (TFAP2E) is related to 5-FU resistance. Highly expressed miR-106a-5p and miR-421 regulate the drug resistance induced by TFAP2E methylation, and bioinformatics analysis predicts that E2F1, MTOR and STAT3 may be target genes. Therefore, sEV- and miRNA-related mechanisms may be used to reverse drug resistance.

Additionally, sEVs may be involved in resistance to doxorubicin [107, 108], vincristine [109] and paclitaxel [110]. However, some data seem to be from cell research and it is speculated that they may be found in sEVs as they belong to a group of molecules which are frequently found in sEVs. Therefore, more rigorous and standardized experiments are needed for verification.

### sEVs and the microenvironment & immunotherapy

**Fig. 3:**
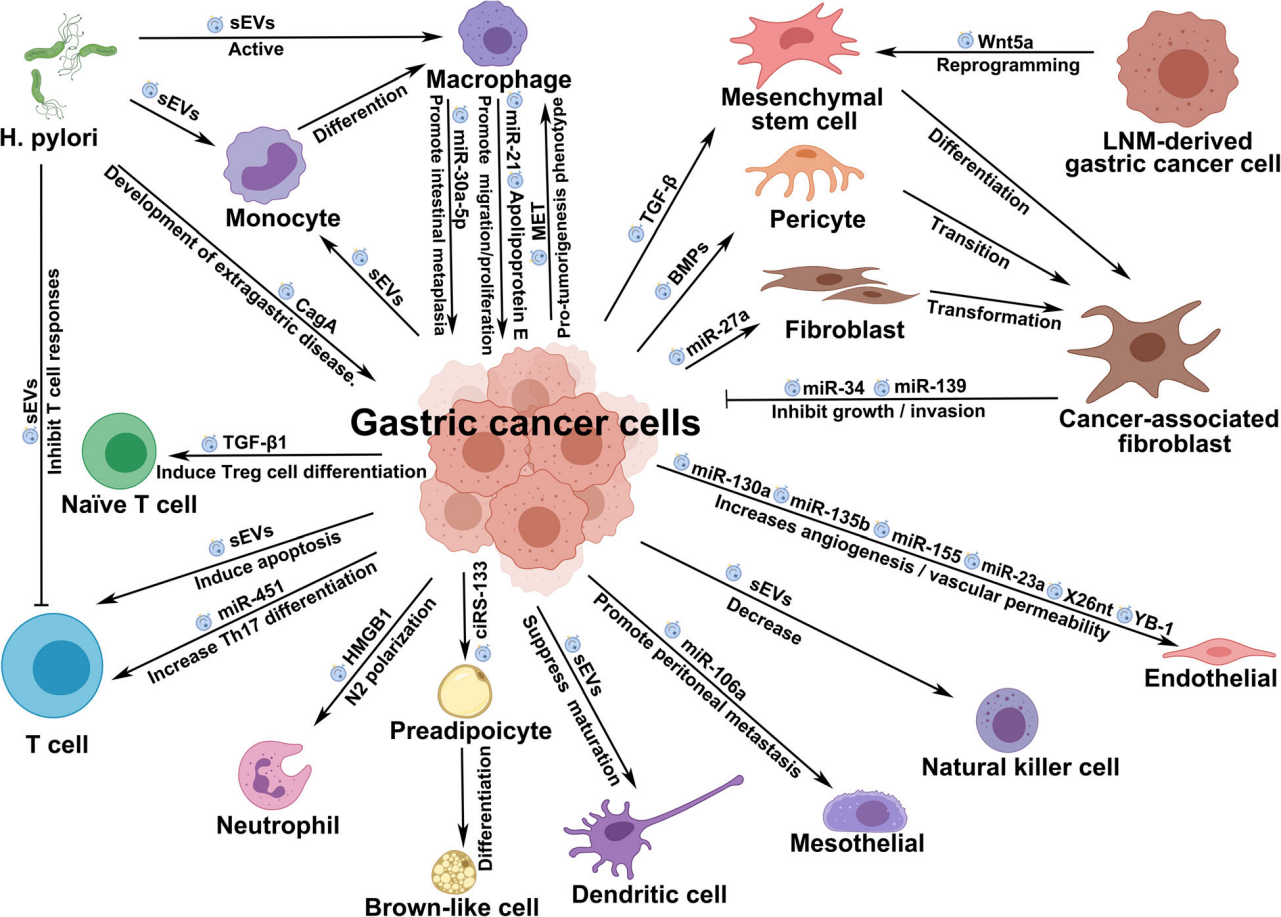
The network of sEVs in GC microenvironment. Abbreviations: sEVs: small extracellular vesicles; LNM: Lymph node metastasis; *H. pylori, Helicobacter pylori*

EVs can also affect the tumour microenvironment, which mainly consists of inflammatory cells, stromal cells, extracellular matrix and so on (Table 4) (Fig. 3). Tumour-associated fibroblasts (CAFs) have a vital role in tumour progression. A recent study revealed that GOF p53-containing sEVs can promote fibroblast transformation into a tumour-associated phenotype [127]. Pericytes retain the characteristics of progenitor cells and differentiate into fibroblasts under pathological conditions. EVs derived from GC cells have been shown to enhance the proliferation and migration of pericytes and induce the expression of CAF markers. The above process is affected by BMP metastasis mediated by sEVs [112]. *Helicobacter pylori* is one of the most important pathogenic factors of antral GC, and CagA encoded by cytotoxin-related genes is the most critical toxin of this bacterium [128]. It has been reported that sEVs secreted by gastric epithelial cells expressing CagA may enter the circulation, deliver CagA to distant organs and tissues, and participate in the development of extragastric diseases related to cagA-positive *H. pylori* infection [113].

**Table 4:** Role of sEVs cargo in the microenvironment of GC

Type	Contents	Donor cells	Recipient cells	Function	Ref.
Protein	HMGB1	BGC	Neutrophils	Induce N2 polarization of neutrophils	[50]
Protein	BMPs	SGC-7901	Pericytes	Induce transition into cancer-associated fibroblasts	[112]
Protein	CagA	CagA-expressing WT-A10	WT-10 and AGS	Involved in the development of extra-gastric disorders associated with CagA-positive *H. pylori* infection	[113]
Protein	Apolipoprotein E	TAM	MFC and MGC- 803	Promote cell migration	[114]
Protein	MET	*H. pylori*-infected AGS	TAM	Educate macrophages towards a pro-tumourigenesis phenotype	[115]
Protein	TGF-β1	Plasma from GC patients	CD4^+^CD45RA^+^ naïve T cells	Induce Treg cell differentiation	[116]
Protein	TGF-β	SGC-7901 and HGC-27	HucMSCs	Trigger differentiation to carcinoma-associated fibroblasts	[117]
miRNA	miR-21	TAM	BGC-823	Contribute to cell proliferation	[118]
miRNA	miR-451	MKN45	Infiltrated T cells	Increase Th17 differentiation	[119]
miRNA	miR-27a	SGC-7901	CCC-HSF-1	Promote transformation into cancer-associated fibroblasts	[120]
miRNA	miR-106a	AGS	Mesothelial cells	Promote peritoneal metastasis	[121]
miRNA	miR-34	Cancer-associated fibroblasts	AGS, AZ521, MKN1, and NUGC3	Inhibit growth and invasion of GC cells	[122]
miRNA	miR-139	Cancer-associated fibroblasts	AGS	Inhibit GC progression	[123]
miRNA	miR-30a-5p	deoxycholic acid-induced macrophage	GES-1	Promote intestinal metaplasia and suppress proliferation	[124]
miRNA	miR-16-5p	M1 macrophages	T cells	Inhibit GC progression	[125]
circRNA	ciRS-133	SGC-7901	3T3L1	Promote differentiation into brown-like cells	[126]

The main component of the tumour microenvironment, namely, tumour-associated macrophages (TAMs), has a tendency to promote cancer. Apolipoprotein E (Apo E) is a highly specific protein secreted by M2 macrophages. Apo E is expressed in the microenvironment of GC and activates the PI3K-Akt signalling pathway to promote metastasis [114]. *H. pylori* also activates MET and has a protumorigenic effect on TAMs [115]. However, the delivery of sEVs from TAMs contributes to cell proliferation [118]. M1 macrophage-derived sEVs containing miR-16-5p were found to trigger a T cell immune response by decreasing the expression of PD-L1, which could eventually suppress tumour progression [125].

Furthermore, sEVs affect the differentiation of T cells [116, 119] and promote transformation to carcinoma-associated fibroblasts [117, 120] and brown-like adipocytes [126]. By targeting Smad7 in peritoneal mesothelial cells, sEV-miR-106a plays a crucial role in GC peritoneal metastasis [121].

After coculture of tumour-associated fibroblasts with GC cells, the proliferation and invasion of the latter were inhibited, indicating that miRNA-34 may have tumour-suppressor effects, but the potential target genes and mechanisms need to be determined [122]. MiR-139, which is also derived from tumour-associated fibroblasts, can inhibit the progression and metastasis of GC by negatively regulating MMP11 [123].

In addition, bile acid might participate in the process of intestinal metaplasia and gastric epithelial cell proliferation [129], but the mechanism has not been fully elucidated. Xu et al. [124] demonstrated that DCA-activated macrophages could secrete sEVs to transport miR-30a-5 to GES-1 cells, thereby affecting the above process by targeting FOXD1. Then, tumour angiogenesis might be induced via the AKT/NF-κB pathway [130].

Finally, some studies have shown the influence of sEVs in the microenvironment, but specific substances have yet to be discovered, and further investigations are needed. Hinata et al. [131] reported that sEVs secreted by Epstein-Barr virus (EBV)-associated gastric cancer cells could suppress dendritic cell maturation, thereby negatively affecting the induction of tumour immunity. Liu et al. [132] showed that GC-derived sEVs could block the cell cycle and induce CD8^+^ T cell apoptosis. Lung metastasis could also be promoted due to the decrease in CD8^+^ T cells and NK cells and the increase in CD4^+^ T cells and myeloid-derived suppressor cells (MDSCs). Exosome-mediated metabolic reprogramming should also be further exploited to elucidate the mechanism of GC progression [133].

sEVs have strong potential in the field of cancer immunotherapy [134]. In clinical practice, with the development of PD-1/PD-L1-related research, immunotherapy-related clinical trials have gradually been carried out in patients with MSI-H and dMMR. Tumour immunotherapy has recently been a hot research topic, with a focus on clinical research related to programmed death receptor protein 1 (PD-1) and programmed death-ligand 1 (PD-L1) inhibitors, mainly for the activation and promotion of immune cells to counteract immune suppression and promote the killing effect against tumours [135]. Previous research has mainly focused on the role of soluble PD-L1, whereas there are few studies on sEV-PD-L1. Due to the secretory properties of sEVs, they can both inhibit and kill T cells in the local tumour microenvironment and be transferred to a remote site to exert other functions, which may be a powerful factor leading to tumour immune escape [136]. Fan et al. reported that due to its stability and T cell dysfunction caused by MHC-I expression, sEV-PD-L1 can reflect the immune status and predict the long-term prognosis of patients [137]. In addition, a study published in 2020 by Zhang et al. [138] stated that the use of 5-FU may upregulate the expression of sEV-PD-L1, which is likely to cause immunosuppression after more than two cycles of chemotherapy. This discovery may affect future comprehensive treatment strategies for advanced GC. It is also expected that further in-depth studies will clarify the relationship between different drug treatments and provide patients with accurate and efficient comprehensive treatment plans.

Most of the related literature has been published in the past three years, which may be due to the rapid development of proteomics and transcriptomics. With the continuous maturity of such technology, it is believed that more tumour-associated effects of sEV contents will be discovered. Of course, the proportion of tumour-associated cargo and the kind of effect sEVs produce under the action of multiple contents remain open questions.

## Application prospects of sEVs in GC

### Diagnosis and prognostic evaluation of GC

The lipid bilayer structure of sEVs protects their cargo from degradation while maintaining a fairly constant content. The noninvasive nature, possibility for real-time assessment and stable characteristics make sEVs an ideal potential biomarker [139, 140]. An sEVs-RNA-based test for early prostate cancer detection has been included in the National Comprehensive Cancer Network (NCCN) guidelines. Indeed, an increasing number of studies have proven that sEVs have strong developmental potential and application prospects in the early diagnosis and prognostic evaluation of GC (Table 5).

**Table 5:** Application of sEVs cargo in diagnosis and prognostic evaluation of GC

Application	Type	Biofluids	Contents	Ref.
Diagnosis of GC	miRNA	Plasma	miR-185, miR-20a, miR-210, miR-25, miR-92b	[141]
	Protein	Serum	GKN1	[55]
	Protein	Serum	MT1-MMP	[142]
	miRNA	Serum	miR10b-5p, miR195-5p, miR20a-3p, miR296-5p	[143]
	lncRNA	Plasma	CEBPA-AS1	[144]
	lncRNA	Serum	HOTTIP	[145]
	lncRNA	Serum	GNAQ-6	[146]
	miRNA	Serum	miR-200a-3p, miR-296-5p, miR-132-3p, miR-485-3p, miR-22-5p	[147]
	miRNA	Plasma	miR-217	[148]
	miRNA	Serum	miR-92a-3p	[149]
	miRNA	Serum	miR-590-5p	[150]
	lncRNA	Serum	pcsk2–2:1	[151]
	miRNA	Serum	miR-19b-3p, miR-106a-5p	[152]
	lncRNA	Plasma	LINC00152	[153]
	lncRNA	Serum	ZFAS1	[65]
Diagnosis of early GC	Protein	gastric juice	BARHL2	[154]
	lncRNA	Serum	GC1	[155]
	miRNA	Serum	miR-92b-3p, let-7 g-5p, miR-146b-5p, miR-9-5p	[156]
	miRNA	Serum	miR-1246	[157]
	circRNA	Plasma	circ-0065149	[158]
Diagnosis of early GC and premalignant chronic atrophic gastritis	lncRNA	Plasma	UEGC1	[159]
Distinguish GC patients with various kinds of metastasis	miRNA	Plasma	miR-10b-5p, miR-101-3p, miR-143-5p	[160]
Diagnosis of malignant GC-associated ascites	miRNA	Ascites	miR-181b-5p	[161]
Predict the recurrence risk and prognosis of patients with GC in each stage.	miRNA	Plasma	miR-23b	[162]
Predict the prognosis of patients with GC	miRNA	Serum	miR-423-5p	[61]
	miRNA	Serum	miR-451	[119]
	lncRNA	Serum	HOTTIP	[145]
	circRNA	Plasma	circ-KIAA1244	[163]
	circRNA	Plasma	circ-0065149	[158]
	circRNA	Plasma	circ-SLC2A12–10:1	[164]
	lncRNA	Serum	MIAT	[165]
Predict the prognosis of GC and risk of lymphatic metastasis	lncRNA	Serum	ZFAS1	[65]
	Protein	Serum	MT1-MMP	[142]
Correlate with tumour stage, lymphatic and distal metastasis, venous and perineural invasion.	circRNA	Serum	circ-0000419	[166]
Predict postoperative haematogenous metastasis of stage II/III GC	miRNA	Serum	miR-379-5p, miR-410-3p	[167]
Predict the peritoneal metastases	miRNA	Ascites	miR-21-5p, miR-92a-3p, miR-223-3p, miR-342-3p	[168]

The earliest high-quality study was conducted by Zhou et al. [141] in 2015. Five highly expressed microRNAs were selected and assessed in the plasma of 30 GC patients and 30 healthy people, with verification in 71 and 61 and areas under the receiver operating characteristic (ROC) curve (AUC) of 0.86 and 0.74, respectively. In the subsequent external verification phase, the AUC was also as high, at 0.87. Unfortunately, a small number of patients do not show any increase in sEV microRNAs, and it may be necessary to further improve the selection of markers. In serum, high expression of membrane type 1 matrix metalloproteinase (MT1-MMP) is considered to be related to the tumour diameter, depth of invasion and TNM stage in GC. Moreover, the sEV-MT1-MMP axis has been proven to be an independent risk factor for GC lymphatic metastasis, which provides strong evidence for serological sEVs to predict the risk of lymphatic metastasis [142]. Huang et al. [143] adopted the Exiqon panel based on qRT-PCR and found 58 differentially expressed miRNAs using three GC sample banks and a normal control sample bank, and six miRNAs in serum (miR10b-5p, miR132-3p, miR185-5p, miR195-5p, miR-20a3p and miR296-5p) were selected for diagnosing GC. Other studies have also reported many new biomarkers [144–153].

In some studies, protein or DNA is the focus of sEVs detection. Yamamoto et al. [154] conducted methylation detection using sEVs derived from GC cell lines, GC tissues and gastric juice and found higher levels of BARHL2 methylation in gastric juice from early GC patients and GC cell lines, with lower levels in normal and atrophic gastritis. Moreover, after resection of early GC via endoscopy, the methylation level was significantly reduced. The AUC of this meaningful study was 0.923, and this approach is expected to become a highly accurate method for detecting early GC. For patients with metastatic GC, Ding et al. [169] provided a comprehensive description of the serum sEVs-proteome and found that several subunits might act as biomarkers and therapeutic targets.

In addition, lncRNAs and circRNAs have become important research molecules in recent years. In July 2020, a multistage study of 522 patients with GC, 85 patients with precancerous lesions of the stomach and 219 healthy people was published [155]. Subsequently, lncRNA-GC1 was found to be significantly elevated in GC cell culture medium, as confirmed in those with precancerous lesions and healthy people. As lncRNA-GC1 is almost exclusively secreted by sEVs, the level of lncRNA-GC1 can remain stable after ribonuclease treatment, even after prolonged exposure to room temperature or repeated freezing and thawing. Various studies have shown that this molecule is likely to become an ideal noninvasive GC diagnostic biomarker. In the future, the combination of lncRNA-GC1 detection and gastroscopy will substantially improve the early diagnosis rate of GC. Zhao et al. [145] analysed serological sEVs from 246 subjects and found that the expression level of HOTTIP in GC was high, with a higher diagnostic ability than CEA, CA 19–9 and CA72–4 (*P* < 0.001). Similarly, lncUEGC1 in sEVs has been confirmed to have a high sensitivity in the diagnosis of GC, which is expected to promote early GC screening in the future [159]. Other studies have contributed to the early diagnosis of GC [156–158].

Zhang et al. [160] used second-generation sequencing technology to obtain the RNA sequencing spectrum of GC patients for the first time and reported that miR-10b-5p, miR-101-3p and miR-143-5p could be used to distinguish lymphatic metastasis, ovarian metastasis and liver metastasis of GC. Such an early and accurate predictive method is conducive to the examination and evaluation of distant organs by clinicians as well as to the early detection of metastatic lesions, which is of practical value. By screening several microRNAs differentially expressed in GC patients and controls followed by further analysis and screening, researchers can use multiple microRNAs in combination for diagnosis and prognostic evaluation. From the perspective of GC ascites identification, Yun et al. used a microarray to detect the expression level of miRNAs in 73 cases of liver cirrhosis-related ascites and 92 cases of gastric cancer-related ascites and found that miR-181b-5p was superior to CEA in the diagnosis of GC ascites. When the two were used in combination, notable areas under the curve of 0.981 and 0.946 were achieved in the training and validation groups, respectively, revealing an excellent method of distinguishing the properties of ascites [161]. Kumata et al. [162] elaborated on the role of sEV-miRNAs in predicting the recurrence of GC: using miRNA chips, they found that miR-23b is specific for recurrence, and its ability to predict recurrence and prognosis at various tumour stages was fully demonstrated in 232 GC patients and 20 healthy volunteers.

Benefiting from their stable properties, sEV containing circRNAs have achieved remarkable results as biomarkers [158, 163–166]. CircRNA expression profile analysis of the plasma from GC patients at different TNM stages and healthy people showed lower circ-KIAA1244 in GC tissues, plasma and cell lines [163]. Through further clinical data analysis, it was found that decreased expression of circ-KIAA1244 correlated negatively with TNM staging and lymphatic metastasis, and the overall survival of such patients was significantly shortened. Subsequently, circ-0000419 was reported to be significantly related to tumour staging, distant metastasis, and venous and nerve infiltration. Both of them may become powerful indicators for early GC screening and prognostic evaluation of advanced GC. The risk of postoperative haematogenous metastasis of stage II/III GC [167] and peritoneal metastases [168] might also be predicted.

In ongoing clinical trials, sEVs are considered biomarkers of cancer diagnosis and prognosis. By monitoring changes in sEVs-HSP70 in the circulation, tumour response and clinical outcome could be predicted (NCT02662621 [170],). There are also two clinical trials focusing on the possibility of circulating sEVs as prognostic markers in advanced GC (NCT01779583) and digestive cancer (NCT04530890) without posted results.

Studies of sEVs as molecular markers are ongoing, and their biological characteristics contribute to the theoretical basis of clinical applications. Several websites have been developed to exhibit potential biomarkers for cancer diagnosis and prognosis [18, 171]. However, a literature review can reveal that early detection technology is dated and expensive, resulting in the small scale of the research to date [139]. As such, sample selection bias is impossible to avoid, which leads to uneven results. In recent years, with the government’s support for big-data platforms, the continuous upgrading of detection technologies and the gradual decline in detection costs, an increasing number of large-sample sequencing studies have been carried out to promote the implementation of sEV biomarkers in clinical practice.

### Cancer drug delivery, antitumour vaccines and engineered sEVs

Efficient delivery and targeting of therapeutic cargo need be achieved to combat tumours, and sEVs can be used as drug delivery vehicles due to their unique physiochemical characteristics, high bioavailability and low nontargeted cytotoxicity [172].

Proton pump inhibitors (PPIs) are commonly used drugs to inhibit gastric acid secretion, and their antitumour potential is gradually being discovered. Guan et al. [173] found that high-dose PPIs can inhibit the release of sEVs and regulate the HIF-1α-FOXO1 axis, thereby regulating the tumour microenvironment and improving the prognosis of patients with advanced GC.

In the present literature, cancer cell-derived sEVs could preferentially fuse with their parent cancer cells [174]. Many sEVs have been used as carriers for antitumour proteins or microRNA inhibitors because of their tissue penetration, nonimmunogenicity, and cell tropism [175]. At present, only two clinical trials have explored the drug-carrying capacity of natural EVs, and they have not produced the expected results (NCT01294072 and NCT01854866). However, the number of patents based on EVs is still low; thus, further research is still warranted [176].

sEVs, especially exosomes, have now entered vaccine development [177, 178]. Numerous studies have shown that tumour-derived exosomes (TEXs) could have immunosuppressive effects as well as immunostimulatory effects [179–181]. Antitumour vaccines based on sEVs have been used to suppress tumour growth through dendritic cell-released MHC class I/peptide complexes for efficient CD8^+^ T cell priming [182]. Cheng et al. also demonstrated that sEVs derived from M1-polarized macrophages have the potential to be used as vaccine adjuvants due to their ability to home to lymph nodes and the expression of proinflammatory T helper cell type 1 cytokines [183]. For exploration of the feasibility of exosomes as immunotherapeutic vaccines, several clinical trials have been carried out or will be carried out (NCT01159288, NCT01550523 and NCT03608631). However, application of these treatments in GC is still progressing slowly.

Although natural EVs can elicit antitumour activities, there are still several problems. Accurate dose monitoring and targeted biodistribution in vivo are difficult to realize due to the high heterogeneity and complicated components of natural EVs, which lead to reduced therapeutic efficacy and safety concerns [184]. Therefore, artificial engineered sEVs have been generated through genetic and chemical methods to improve antitumour efficacy [185, 186].

Various strategies have been used for sEV engineering, of which loading target cargo or modification are two major strategies [187]. Cargo active loading into donor cells and direct loading into sEVs are the main components of the former [188]. In addition to constantly developing general modifications of EV membranes [189], new engineered EV-based platforms, such as EV-mimetic nanovesicles [190] and artificially synthesized EV-like synthetic nanoparticles [191], have gradually been developed.

There have been several animal models to investigate the utility of EVs for cancer drug delivery. For gastric cancer, sEVs containing anti-miR-214 could strengthen chemosensitivity and inhibit tumour growth in a cisplatin-resistant gastric cancer mouse model [52]. Pan et al. demonstrated that PMA/Au-BSA@Ce6 nanoparticles with deep penetration and superior retention performance could be used for cancer-targeted photodynamic therapy [192]. Genetically engineered K562-secreted sEVs containing HLA-A2 and several costimulatory molecules could activate CD8^+^ T cells to strengthen the effectiveness of immunotherapy [193]. Unfortunately, there is no research on sEVs in the chemotherapy of gastric cancer.

**Fig. 4:**
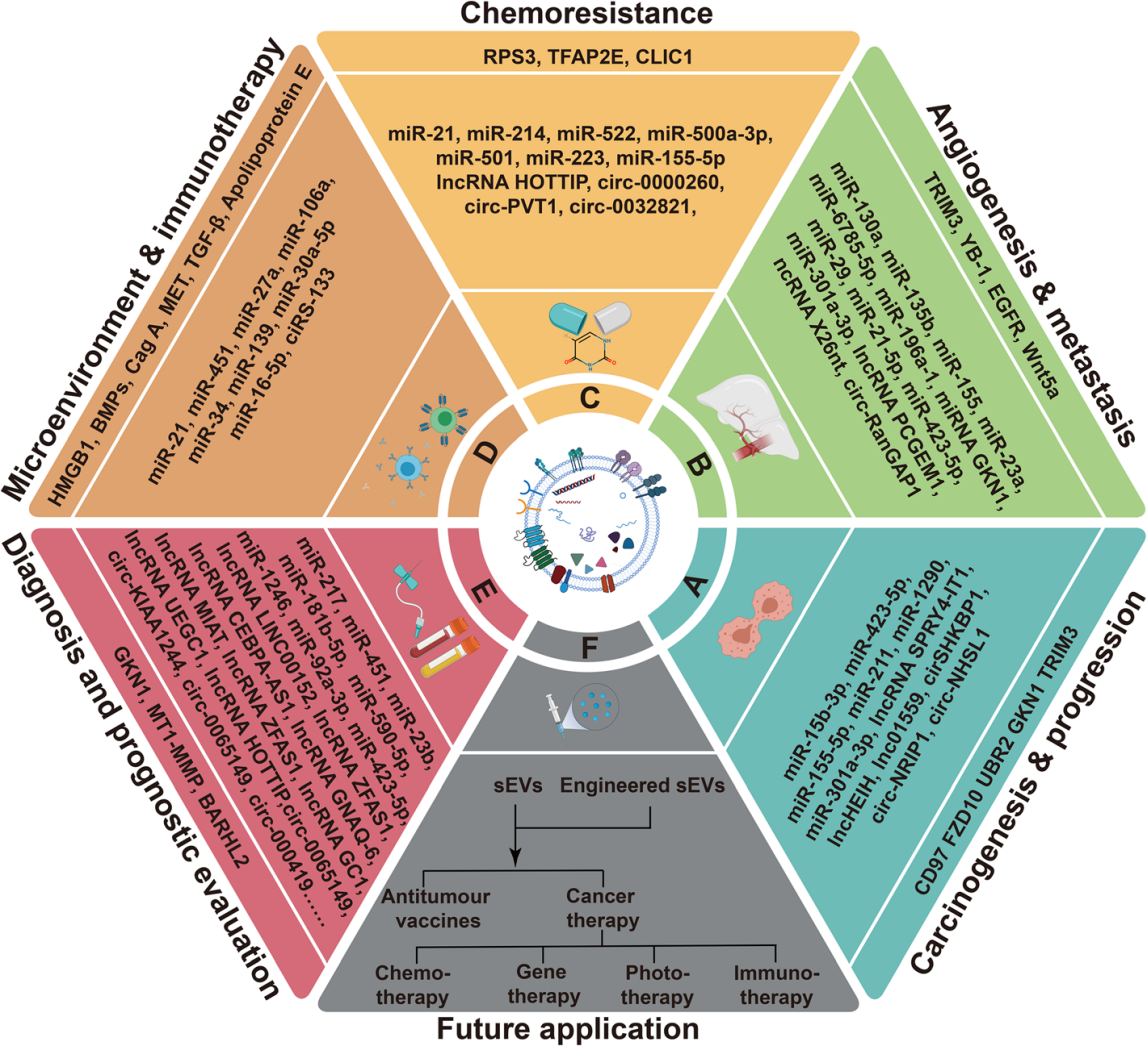
sEVs play a significant role in gastric cancer carcinogenesis and progression (**a**), angiogenesis and metastasis (**b**), chemoresistance (**c**), microenvironment and immunotherapy (**d**). sEVs can be applicated in diagnosis and prognostic evaluation (**e**), and other future aspects (**f**)

As shown above, although the application of sEVs has been extensively and profoundly explored in many cancers, further studies on this topic in gastric cancer are needed. We drew a compass-shaped diagram to show the role and application of sEVs in gastric cancer intuitively (Fig. 4). It is urgent to advance our understanding of sEVs to unlock their full potential in the prevention and treatment of gastric cancer. In addition, it also urgent to set up reliable assays to assess the therapeutic potential of sEVs, and to develop such assays into formal potency tests for cinical application [194].

## Conclusion

Extensive attention has been paid to EVs in recent years. This research expands our understanding of the functions of sEVs and the mechanism of tumorigenesis and provides a new perspective for the prevention and treatment of gastric cancer. The mechanism of action of gastric cancer-related sEVs mainly depends on their complex cargo. In addition, communication between GC cells and the tumour microenvironment, as well as many unknown mechanisms and molecules, adds to the overall complexity and uncertainty.

Of course, opportunities and challenges coexist. The complicated cargo emphasizes the complexity of the mechanism, and the dominant roles of the various molecules and the mechanisms of the comprehensive effect are still unknown. Limited by immature technology, the diversity of detection methods and results, and the high cost of detection, few large-sample, multicentre studies have been conducted to date. Moreover, the genetic differences between East and West, between countries, and even between regions are likely to challenge the universality of existing research. Thus, prior to the clinical application of scientific results, there is an urgent need for further evaluation of the safety, effectiveness and stability of sEVs, and clinicians, pharmacists, and other professional scientists need to work together. Regardless, it is foreseeable that due to their unique biological characteristics, sEVs will become an important tool in the near future for accurate early diagnosis and personalized and efficient treatment of tumours, providing infinite power to overcome cancers.

## Abbreviations

GC: Gastric cancer
sEVs: small extracellular vesicles
EVs: Extracellular vesicles
MISEV: Minimal Information for Studies of Extracellular Vesicles
ESCRT: Endosomal sorting complex required for transport
miRNAs: microRNA
circRNA: circular RNA
lncRNA: long noncoding RNA
MVB: Multivesicular bodies
MSC: Mesenchymal stem cell
CAF: Cancer-associated fibroblast
EMT: Epithelial mesenchymal transition
*H. pylori*: *Helicobacter pylori*
TAM: Tumour-associated macrophage
TNF-α: Tumour necrosis factor alpha
GMCSF: Granulocyte-macrophage colony stimulating factor
IL: Interleukin
PMC: Peritoneal mesothelial cell
5-FU: 5-Fluorouracil
HUVESs: Human umbilical vein endothelial cells
GKN1: Gastrokine 1
FZD10: Frizzled 10
UBR2: N-recognin 2
MMT: Mesothelial-to-mesenchymal transition
AUC: Area under curve
ROC: Receiver operating characteristic
MT1-MMP: Membrane type 1 matrix metalloproteinase
ALOX15: Arachidonic acid lipoxygenase 15
TFAP2E: Transcription factor activation promoter binding protein 2e
Apo E: Apolipoprotein E
MDSC: Myeloid-derived suppressor cell
PD-1: Programmed death receptor protein 1
PD-L1: Programmed death-ligand 1
PPI: Proton pump inhibitors
NCCN: National Comprehensive Cancer Network

## References

[1] Sung H, Ferlay J, Siegel RL, Laversanne M, Soerjomataram I, Jemal A, et al. Global cancer statistics 2020: GLOBOCAN estimates of incidence and mortality worldwide for 36 cancers in 185 countries. CA Cancer J Clin. 2021. 10.3322/caac.21660 33538338

[2] Fu M, Gu J, Jiang P, Qian H, Xu W, Zhang X (2019). Exosomes in gastric cancer: roles, mechanisms, and applications. Mol Cancer.

[3] Huang T, Song C, Zheng L, Xia L, Li Y, Zhou Y (2019). The roles of extracellular vesicles in gastric cancer development, microenvironment, anti-cancer drug resistance, and therapy. Mol Cancer.

[4] Thery C, Witwer KW, Aikawa E, Alcaraz MJ, Anderson JD, Andriantsitohaina R, Antoniou A, Arab T, Archer F, Atkin-Smith GK (2018). Minimal information for studies of extracellular vesicles 2018 (MISEV2018): a position statement of the International Society for Extracellular Vesicles and update of the MISEV2014 guidelines. J Extracell Vesicles.

[5] Pan BT, Johnstone RM (1983). Fate of the transferrin receptor during maturation of sheep reticulocytes in vitro: selective externalization of the receptor. Cell.

[6] Doyle LM, Wang MZ. Overview of extracellular vesicles, their origin, composition, purpose, and methods for exosome isolation and analysis. Cells. 2019;8:727. 10.3390/cells8070727 PMC6678302 31311206

[7] Borges FT, Reis LA, Schor N (2013). Extracellular vesicles: structure, function, and potential clinical uses in renal diseases. Braz J Med Biol Res.

[8] Gould SJ, Booth AM, Hildreth JE (2003). The Trojan exosome hypothesis. Proc Natl Acad Sci U S A.

[9] Fang Y, Wu N, Gan X, Yan W, Morrell JC, Gould SJ (2007). Higher-order oligomerization targets plasma membrane proteins and HIV gag to exosomes. PLoS Biol.

[10] Tian X, Shen H, Li Z, Wang T, Wang S (2019). Tumor-derived exosomes, myeloid-derived suppressor cells, and tumor microenvironment. J Hematol Oncol.

[11] Wei D, Zhan W, Gao Y, Huang L, Gong R, Wang W, et al. RAB31 marks and controls an ESCRT-independent exosome pathway. Cell Res. 2020;31:157–77. 10.1038/s41422-020-00409-1 PMC8027411 32958903

[12] Frydrychowicz M, Kolecka-Bednarczyk A, Madejczyk M, Yasar S, Dworacki G (2015). Exosomes - structure, biogenesis and biological role in non-small-cell lung cancer. Scand J Immunol.

[13] Pegtel DM, Gould SJ (2019). Exosomes. Annu Rev Biochem.

[14] Cocucci E, Meldolesi J (2015). Ectosomes and exosomes: shedding the confusion between extracellular vesicles. Trends Cell Biol.

[15] Atkin-Smith GK, Tixeira R, Paone S, Mathivanan S, Collins C, Liem M, Goodall KJ, Ravichandran KS, Hulett MD, Poon IK (2015). A novel mechanism of generating extracellular vesicles during apoptosis via a beads-on-a-string membrane structure. Nat Commun.

[16] Li Y, Zhao J, Yu S, Wang Z, He X, Su Y, Guo T, Sheng H, Chen J, Zheng Q (2019). Extracellular vesicles Long RNA sequencing reveals abundant mRNA, circRNA, and lncRNA in human blood as potential biomarkers for Cancer diagnosis. Clin Chem.

[17] Makarova J, Turchinovich A, Shkurnikov M, Tonevitsky A. Extracellular miRNAs and cell-cell communication: problems and prospects. Trends Biochem Sci. 2021. 10.1016/j.tibs.2021.01.007 33610425

[18] Wang S, Zhang K, Tan S, Xin J, Yuan Q, Xu H, Xu X, Liang Q, Christiani DC, Wang M (2021). Circular RNAs in body fluids as cancer biomarkers: the new frontier of liquid biopsies. Mol Cancer.

[19] Sun Z, Yang S, Zhou Q, Wang G, Song J, Li Z, Zhang Z, Xu J, Xia K, Chang Y (2018). Emerging role of exosome-derived long non-coding RNAs in tumor microenvironment. Mol Cancer.

[20] Campos-Carrillo A, Weitzel JN, Sahoo P, Rockne R, Mokhnatkin JV, Murtaza M, Gray SW, Goetz L, Goel A, Schork N, Slavin TP (2020). Circulating tumor DNA as an early cancer detection tool. Pharmacol Ther.

[21] Mathieu M, Martin-Jaular L, Lavieu G, Thery C (2019). Specificities of secretion and uptake of exosomes and other extracellular vesicles for cell-to-cell communication. Nat Cell Biol.

[22] Monguio-Tortajada M, Galvez-Monton C, Bayes-Genis A, Roura S, Borras FE (2019). Extracellular vesicle isolation methods: rising impact of size-exclusion chromatography. Cell Mol Life Sci.

[23] Patel GK, Khan MA, Zubair H, Srivastava SK, Khushman M, Singh S, Singh AP (2019). Comparative analysis of exosome isolation methods using culture supernatant for optimum yield, purity and downstream applications. Sci Rep.

[24] Gardiner C, Di Vizio D, Sahoo S, Thery C, Witwer KW, Wauben M, Hill AF (2016). Techniques used for the isolation and characterization of extracellular vesicles: results of a worldwide survey. J Extracell Vesicles.

[25] Lazaro-Ibanez E, Lasser C, Shelke GV, Crescitelli R, Jang SC, Cvjetkovic A, Garcia-Rodriguez A, Lotvall J (2019). DNA analysis of low- and high-density fractions defines heterogeneous subpopulations of small extracellular vesicles based on their DNA cargo and topology. J Extracell Vesicles.

[26] He M, Crow J, Roth M, Zeng Y, Godwin AK (2014). Integrated immunoisolation and protein analysis of circulating exosomes using microfluidic technology. Lab Chip.

[27] Li P, Kaslan M, Lee SH, Yao J, Gao Z (2017). Progress in Exosome Isolation Techniques. Theranostics.

[28] Kornilov R, Puhka M, Mannerstrom B, Hiidenmaa H, Peltoniemi H, Siljander P, Seppanen-Kaijansinkko R, Kaur S (2018). Efficient ultrafiltration-based protocol to deplete extracellular vesicles from fetal bovine serum. J Extracell Vesicles.

[29] Dong L, Zieren RC, Horie K, Kim CJ, Mallick E, Jing Y, Feng M, Kuczler MD, Green J, Amend SR (2020). Comprehensive evaluation of methods for small extracellular vesicles separation from human plasma, urine and cell culture medium. J Extracell Vesicles.

[30] Yang D, Zhang W, Zhang H, Zhang F, Chen L, Ma L, Larcher LM, Chen S, Liu N, Zhao Q (2020). Progress, opportunity, and perspective on exosome isolation - efforts for efficient exosome-based theranostics. Theranostics.

[31] Crescitelli R, Lasser C, Lotvall J. Isolation and characterization of extracellular vesicle subpopulations from tissues. Nat Protoc. 2021;16:1548–80. 10.1038/s41596-020-00466-1 33495626

[32] Wu Y, Zhang N, Wu H, Sun N, Deng C (2021). Magnetic porous carbon-dependent platform for the determination of N-glycans from urine exosomes. Mikrochim Acta.

[33] Lin Q, Huang Z, Ye X, Yang B, Fang X, Liu B, Chen H, Kong J (2021). Lab in a tube: isolation, extraction, and isothermal amplification detection of exosomal long noncoding RNA of gastric cancer. Talanta.

[34] Yang HC, Ham YM, Kim JA, Rhee WJ (2021). Single-step equipment-free extracellular vesicle concentration using super absorbent polymer beads. J Extracell Vesicles.

[35] Booth AM, Fang Y, Fallon JK, Yang JM, Hildreth JE, Gould SJ (2006). Exosomes and HIV gag bud from endosome-like domains of the T cell plasma membrane. J Cell Biol.

[36] Nolan JP, Duggan E (1678). Analysis of individual extracellular vesicles by flow Cytometry. Methods Mol Biol.

[37] Shao H, Im H, Castro CM, Breakefield X, Weissleder R, Lee H (2018). New Technologies for Analysis of extracellular vesicles. Chem Rev.

[38] Witwer KW, Soekmadji C, Hill AF, Wauben MH, Buzas EI, Di Vizio D, Falcon-Perez JM, Gardiner C, Hochberg F, Kurochkin IV (2017). Updating the MISEV minimal requirements for extracellular vesicle studies: building bridges to reproducibility. J Extracell Vesicles.

[39] Mendt M, Kamerkar S, Sugimoto H, McAndrews KM, Wu CC, Gagea M, et al. Generation and testing of clinical-grade exosomes for pancreatic cancer. JCI Insight. 2018;3:e99263. 10.1172/jci.insight.99263 PMC5931131 29669940

[40] Maroto R, Zhao Y, Jamaluddin M, Popov VL, Wang H, Kalubowilage M, Zhang Y, Luisi J, Sun H, Culbertson CT (2017). Effects of storage temperature on airway exosome integrity for diagnostic and functional analyses. J Extracell Vesicles.

[41] Mashouri L, Yousefi H, Aref AR, Ahadi AM, Molaei F, Alahari SK (2019). Exosomes: composition, biogenesis, and mechanisms in cancer metastasis and drug resistance. Mol Cancer.

[42] Qu JL, Qu XJ, Qu JL, Qu XJ, Zhao MF, Teng YE, Zhang Y, Hou KZ, Jiang YH, Yang XH, Liu YP (2009). The role of cbl family of ubiquitin ligases in gastric cancer exosome-induced apoptosis of Jurkat T cells. Acta Oncol.

[43] Qu JL, Qu XJ, Zhao MF, Teng YE, Zhang Y, Hou KZ, Jiang YH, Yang XH, Liu YP (2009). Gastric cancer exosomes promote tumour cell proliferation through PI3K/Akt and MAPK/ERK activation. Dig Liver Dis.

[44] Liu D, Li C, Trojanowicz B, Li X, Shi D, Zhan C, Wang Z, Chen L (2016). CD97 promotion of gastric carcinoma lymphatic metastasis is exosome dependent. Gastric Cancer.

[45] Li C, Liu DR, Li GG, Wang HH, Li XW, Zhang W, Wu YL, Chen L (2015). CD97 promotes gastric cancer cell proliferation and invasion through exosome-mediated MAPK signaling pathway. World J Gastroenterol.

[46] Tanaka M, Kuriyama S, Itoh G, Maeda D, Goto A, Tamiya Y, Yanagihara K, Yashiro M, Aiba N (2017). Mesothelial cells create a novel tissue niche that facilitates gastric Cancer invasion. Cancer Res.

[47] Wu L, Zhang X, Zhang B, Shi H, Yuan X, Sun Y, Pan Z, Qian H, Xu W (2016). Exosomes derived from gastric cancer cells activate NF-kappaB pathway in macrophages to promote cancer progression. Tumour Biol.

[48] Wang F, Li B, Wei Y, Zhao Y, Wang L, Zhang P, Yang J, He W, Chen H, Jiao Z, Li Y (2018). Tumor-derived exosomes induce PD1(+) macrophage population in human gastric cancer that promotes disease progression. Oncogenesis.

[49] Shen Y, Xue C, Li X, Ba L, Gu J, Sun Z, Han Q, Zhao RC (2019). Effects of gastric Cancer cell-derived Exosomes on the immune regulation of Mesenchymal stem cells by the NF-kB signaling pathway. Stem Cells Dev.

[50] Zhang X, Shi H, Yuan X, Jiang P, Qian H, Xu W (2018). Tumor-derived exosomes induce N2 polarization of neutrophils to promote gastric cancer cell migration. Mol Cancer.

[51] Ji R, Zhang B, Zhang X, Xue J, Yuan X, Yan Y, Wang M, Zhu W, Qian H, Xu W (2015). Exosomes derived from human mesenchymal stem cells confer drug resistance in gastric cancer. Cell Cycle.

[52] Wang X, Zhang H, Bai M, Ning T, Ge S, Deng T, Liu R, Zhang L, Ying G, Ba Y (2018). Exosomes serve as nanoparticles to deliver anti-miR-214 to reverse Chemoresistance to Cisplatin in gastric Cancer. Mol Ther.

[53] Scavo MP, Depalo N, Rizzi F, Ingrosso C, Fanizza E, Chieti A, et al. FZD10 carried by Exosomes sustains Cancer cell proliferation. Cells. 2019;8:777. 10.3390/cells8080777 PMC6721576 31349740

[54] Mao J, Liang Z, Zhang B, Yang H, Li X, Fu H, Zhang X, Yan Y, Xu W, Qian H (2017). UBR2 enriched in p53 deficient mouse bone marrow Mesenchymal stem cell-exosome promoted gastric Cancer progression via Wnt/beta-catenin pathway. Stem Cells.

[55] Yoon JH, Ham IH, Kim O, Ashktorab H, Smoot DT, Nam SW, Lee JY, Hur H, Park WS (2018). Gastrokine 1 protein is a potential theragnostic target for gastric cancer. Gastric Cancer.

[56] Fu H, Yang H, Zhang X, Wang B, Mao J, Li X, Wang M, Zhang B, Sun Z, Qian H, Xu W (2018). Exosomal TRIM3 is a novel marker and therapy target for gastric cancer. J Exp Clin Cancer Res.

[57] Wei S, Peng L, Yang J, Sang H, Jin D, Li X, Chen M, Zhang W, Dang Y, Zhang G (2020). Exosomal transfer of miR-15b-3p enhances tumorigenesis and malignant transformation through the DYNLT1/Caspase-3/Caspase-9 signaling pathway in gastric cancer. J Exp Clin Cancer Res.

[58] Wang M, Zhao C, Shi H, Zhang B, Zhang L, Zhang X, Wang S, Wu X, Yang T, Huang F (2014). Deregulated microRNAs in gastric cancer tissue-derived mesenchymal stem cells: novel biomarkers and a mechanism for gastric cancer. Br J Cancer.

[59] Ma M, Chen S, Liu Z, Xie H, Deng H, Shang S, Wang X, Xia M (2017). Zuo C: miRNA-221 of exosomes originating from bone marrow mesenchymal stem cells promotes oncogenic activity in gastric cancer. Onco Targets Ther.

[60] Huang J, Shen M, Yan M, Cui Y, Gao Z, Meng X (2019). Exosome-mediated transfer of miR-1290 promotes cell proliferation and invasion in gastric cancer via NKD1. Acta Biochim Biophys Sin Shanghai.

[61] Yang H, Fu H, Wang B, Zhang X, Mao J, Li X, Wang M, Sun Z, Qian H, Xu W (2018). Exosomal miR-423-5p targets SUFU to promote cancer growth and metastasis and serves as a novel marker for gastric cancer. Mol Carcinog.

[62] Shi SS, Zhang HP, Yang CQ, Li LN, Shen Y, Zhang YQ (2020). Exosomal miR-155-5p promotes proliferation and migration of gastric cancer cells by inhibiting TP53INP1 expression. Pathol Res Pract.

[63] Xia X, Wang S, Ni B, Xing S, Cao H, Zhang Z, Yu F, Zhao E, Zhao G (2020). Hypoxic gastric cancer-derived exosomes promote progression and metastasis via MiR-301a-3p/PHD3/HIF-1 ¦Á positive feedback loop. Oncogene.

[64] Cao S, Lin L, Xia X (2019). Wu H: lncRNA SPRY4-IT1 regulates cell proliferation and migration by sponging miR-101-3p and regulating AMPK expression in gastric Cancer. Mol Ther Nucleic Acids.

[65] Pan L, Liang W, Fu M, Huang ZH, Li X, Zhang W, Zhang P, Qian H, Jiang PC, Xu WR, Zhang X (2017). Exosomes-mediated transfer of long noncoding RNA ZFAS1 promotes gastric cancer progression. J Cancer Res Clin Oncol.

[66] Lu Y, Hou K, Li M, Wu X, Yuan S (2020). Exosome-delivered LncHEIH promotes gastric Cancer progression by Upregulating EZH2 and stimulating me thylation of the GSDME promoter. Front Cell Dev Biol.

[67] Wang L, Bo X, Yi X, Xiao X, Zheng Q, Ma L, Li B (2020). Exosome-transferred LINC01559 promotes the progression of gastric cancer via PI3K/AKT signaling pathw ay. Cell Death Dis.

[68] Zhang X, Wang S, Wang H, Cao J, Huang X, Chen Z, Xu P, Sun G, Xu J, Lv J, Xu Z (2019). Circular RNA circNRIP1 acts as a microRNA-149-5p sponge to promote gastric cancer progression via the AKT1/mTOR pathway. Mol Cancer.

[69] Xie M, Yu T, Jing X, Ma L, Fan Y, Yang F, Ma P, Jiang H, Wu X, Shu Y, Xu T (2020). Exosomal circSHKBP1 promotes gastric cancer progression via regulating the miR-582-3p/HUR/VEGF axis and suppressing HSP90 degradation. Mol Cancer.

[70] Hui C, Tian L, He X (2020). Circular RNA circNHSL1 contributes to gastric Cancer progression through the miR-149-5p/YWHAZ Axis. Cancer Manag Res.

[71] Yoon JH, Ashktorab H, Smoot DT, Nam SW, Hur H, Park WS (2020). Uptake and tumor-suppressive pathways of exosome-associated GKN1 protein in gastric epithelial cells. Gastric Cancer.

[72] Zhang J, Qiu WQ, Zhu H, Liu H, Sun JH, Chen Y, Shen H, Qian CL, Shen ZY (2020). HOTAIR contributes to the carcinogenesis of gastric cancer via modulating cellular and exosomal miRNA s level. Cell Death Dis.

[73] Brahimi-Horn MC, Chiche J, Pouyssegur J (2007). Hypoxia and cancer. J Mol Med (Berl).

[74] Qi J, Zhou Y, Jiao Z, Wang X, Zhao Y, Li Y, Chen H, Yang L, Zhu H, Li Y (2017). Exosomes derived from human bone marrow Mesenchymal stem cells promote tumor growth through hedgehog signaling pathway. Cell Physiol Biochem.

[75] Gu H, Ji R, Zhang X, Wang M, Zhu W, Qian H, Chen Y, Jiang P, Xu W (2016). Exosomes derived from human mesenchymal stem cells promote gastric cancer cell growth and migration via the activation of the Akt pathway. Mol Med Rep.

[76] Xue X, Huang J, Yu K, Chen X, He Y, Qi D, Wu Y (2020). YB-1 transferred by gastric cancer exosomes promotes angiogenesis via enhancing the expression of ang iogenic factors in vascular endothelial cells. BMC Cancer.

[77] Zhang H, Deng T, Liu R, Bai M, Zhou L, Wang X, Li S, Wang X, Yang H, Li J (2017). Exosome-delivered EGFR regulates liver microenvironment to promote gastric cancer liver metastasis. Nat Commun.

[78] Wang M, Zhao X, Qiu R, Gong Z, Huang F, Yu W, et al. Lymph node metastasis-derived gastric cancer cells educate bone marrow-derived mesenchymal stem cells via YAP signaling activation by exosomal Wnt5a. Oncogene. 2021;40:2296–308. 10.1038/s41388-021-01722-8 PMC7994201 33654199

[79] Yang H, Zhang H, Ge S, Ning T, Bai M, Li J, Li S, Sun W, Deng T, Zhang L (2018). Exosome-derived miR-130a activates angiogenesis in gastric Cancer by targeting C-MYB in vascular endothelial cells. Mol Ther.

[80] Bai M, Li J, Yang H, Zhang H, Zhou Z, Deng T, Zhu K, Ning T, Fan Q, Ying G (2019). Ba Y: miR-135b delivered by gastric tumor Exosomes inhibits FOXO1 expression in endothelial cells and promotes angiogenesis. Mol Ther.

[81] Deng T, Zhang H, Yang H, Wang H, Bai M, Sun W, Wang X, Si Y, Ning T, Zhang L (2020). Exosome miR-155 derived from gastric carcinoma promotes angiogenesis by targeting the c-MYB/VEGF Axis of endothelial cells. Mol Ther Nucleic Acids.

[82] Zhou Z, Zhang H, Deng T, Ning T, Liu R, Liu D, Bai M, Ying G, Ba Y (2019). Exosomes carrying MicroRNA-155 target Forkhead box O3 of endothelial cells and promote angiogenesis in gastric Cancer. Mol Ther Oncolytics.

[83] Du J, Liang Y, Li J, Zhao JM, Wang ZN, Lin XY (2020). Gastric Cancer cell-derived Exosomal microRNA-23a promotes angiogenesis by targeting PTEN. Front Oncol.

[84] Chen Z, Xie Y, Chen W, Li T, Chen X. Liu B: microRNA-6785-5p-loaded human umbilical cord mesenchymal stem cells-derived exosomes suppress angiogenesis and metastasis in gastric cancer via INHBA. Life Sci. 2021:119222. 10.1016/j.lfs.2021.119222 33609542

[85] Feng C, She J, Chen X, Zhang Q, Zhang X, Wang Y, Ye J, Shi J, Tao J, Feng M (2019). Exosomal miR-196a-1 promotes gastric cancer cell invasion and metastasis by targeting SFRP1. Nanomedicine (London).

[86] Ohzawa H, Saito A, Kumagai Y, Kimura Y, Yamaguchi H, Hosoya Y, Lefor AK, Sata N, Kitayama J (2020). Reduced expression of exosomal miR29s in peritoneal fluid is a useful predictor of peritoneal recurrence after curative resection of gastric cancer with serosal involvement. Oncol Rep.

[87] Li Q, Li B, Li Q, Wei S, He Z, Huang X, Wang L, Xia Y, Xu Z, Li Z (2018). Exosomal miR-21-5p derived from gastric cancer promotes peritoneal metastasis via mesothelial-to-mesenchymal transition. Cell Death Dis.

[88] Piao HY, Guo S, Wang Y, Zhang J. Exosome-transmitted lncRNA PCGEM1 promotes invasive and metastasis in gastric cancer by maintaining the stability of SNAI1. Clin Transl Oncol. 2021;23:246–56. 10.1007/s12094-020-02412-9 32519176

[89] Chen X, Zhang S, Du K, Zheng N, Liu Y, Chen H, et al. Gastric Cancer-secreted Exosomal X26nt increases angiogenesis and vascular permeability by targeting VE-cadherin. Cancer Sci. 2020. 10.1111/cas.14740 PMC8088954 33205567

[90] Lu J, Wang YH, Yoon C, Huang XY, Xu Y, Xie JW, Wang JB, Lin JX, Chen QY, Cao LL (2020). Circular RNA circ-RanGAP1 regulates VEGFA expression by targeting miR-877-3p to facilitate gastric cancer invasion and metastasis. Cancer Lett.

[91] Costa-Silva B, Aiello NM, Ocean AJ, Singh S, Zhang H, Thakur BK, Becker A, Hoshino A, Mark MT, Molina H (2015). Pancreatic cancer exosomes initiate pre-metastatic niche formation in the liver. Nat Cell Biol.

[92] Peinado H, Aleckovic M, Lavotshkin S, Matei I, Costa-Silva B, Moreno-Bueno G, Hergueta-Redondo M, Williams C, Garcia-Santos G, Ghajar C (2012). Melanoma exosomes educate bone marrow progenitor cells toward a pro-metastatic phenotype through MET. Nat Med.

[93] Feng W, Dean DC, Hornicek FJ, Shi H, Duan Z (2019). Exosomes promote pre-metastatic niche formation in ovarian cancer. Mol Cancer.

[94] Moller A, Lobb RJ (2020). The evolving translational potential of small extracellular vesicles in cancer. Nat Rev Cancer.

[95] Hoshino A, Costa-Silva B, Shen TL, Rodrigues G, Hashimoto A, Tesic Mark M, Molina H, Kohsaka S, Di Giannatale A, Ceder S (2015). Tumour exosome integrins determine organotropic metastasis. Nature.

[96] Deng G, Qu J, Zhang Y, Che X, Cheng Y, Fan Y, Zhang S, Na D, Liu Y, Qu X (2017). Gastric cancer-derived exosomes promote peritoneal metastasis by destroying the mesothelial barrier. FEBS Lett.

[97] Lepeltier E, Rijo P, Rizzolio F, Popovtzer R, Petrikaite V, Assaraf YG, Passirani C (2020). Nanomedicine to target multidrug resistant tumors. Drug Resist Updat.

[98] Sun MY, Xu B, Wu QX, Chen WL, Cai S, Zhang H, Tang QF (2021). Cisplatin-resistant gastric Cancer cells promote the Chemoresistance of Cisplatin-sensitive cells via the Exosomal RPS3-mediated PI3K-Akt-Cofilin-1 signaling Axis. Front Cell Dev Biol.

[99] Zheng P, Chen L, Yuan X, Luo Q, Liu Y, Xie G, Ma Y, Shen L (2017). Exosomal transfer of tumor-associated macrophage-derived miR-21 confers cisplatin resistance in gastric cancer cells. J Exp Clin Cancer Res.

[100] Zhang H, Deng T, Liu R, Ning T, Yang H, Liu D, Zhang Q, Lin D, Ge S, Bai M (2020). CAF secreted miR-522 suppresses ferroptosis and promotes acquired chemo-resistance in gastric cancer. Mol Cancer.

[101] Lin H, Zhang L, Zhang C, Liu P. Exosomal MiR-500a-3p promotes cisplatin resistance and stemness via negatively regulating FBXW7 in gastric cancer. J Cell Mol Med. 2020;24:8930–41. 10.1111/jcmm.15524 PMC7417713 32588541

[102] Wang J, Lv B, Su Y, Wang X, Bu J, Yao L (2019). Exosome-mediated transfer of lncRNA HOTTIP promotes Cisplatin resistance in gastric Cancer cells by regulating HMGA1/miR-218 Axis. Onco Targets Ther.

[103] Liu S, Wu M, Peng M (2020). Circ_0000260 regulates the development and deterioration of gastric adenocarcinoma with Cisplatin res istance by Upregulating MMP11 via targeting MiR-129-5p. Cancer Manag Res.

[104] Yao W, Guo P, Mu Q, Wang Y. Exosome-derived Circ-PVT1 contributes to Cisplatin resistance by regulating autophagy, invasion, and apoptosis via miR-30a-5p/YAP1 Axis in gastric Cancer cells. Cancer Biother Radiopharm. 2020. 10.1089/cbr.2020.3578 32799541

[105] Zhong Y, Wang D, Ding Y, Tian G, Jiang B. Circular RNA circ_0032821 contributes to oxaliplatin (OXA) resistance of gastric cancer cells by regulating SOX9 via miR-515-5p. Biotechnol Lett. 2020;43:339–51. 10.1007/s10529-020-03036-3 33123829

[106] Jingyue S, Xiao W, Juanmin Z, Wei L, Daoming L, Hong X (2019). TFAP2E methylation promotes 5fluorouracil resistance via exosomal miR106a5p and miR421 in gastric cancer MGC803 cells. Mol Med Rep.

[107] Liu X, Lu Y, Xu Y, Hou S, Huang J, Wang B, Zhao J, Xia S, Fan S, Yu X (2019). Exosomal transfer of miR-501 confers doxorubicin resistance and tumorigenesis via targeting of BLID in gastric cancer. Cancer Lett.

[108] Gao H, Ma J, Cheng Y, Zheng P (2020). Exosomal transfer of macrophage-derived miR-223 confers doxorubicin resistance in gastric Cancer. Onco Targets Ther.

[109] Zhao K, Wang Z, Li X, Liu JL, Tian L, Chen JQ (2019). Exosome-mediated transfer of CLIC1 contributes to the vincristine-resistance in gastric cancer. Mol Cell Biochem.

[110] Wang M, Qiu R, Yu S, Xu X, Li G, Gu R, Tan C, Zhu W, Shen B (2019). Paclitaxelresistant gastric cancer MGC803 cells promote epithelialtomesenchymal transition and chemoresistance in paclitaxelsensitive cells via exosomal delivery of miR1555p. Int J Oncol.

[111] Stockwell BR, Friedmann Angeli JP, Bayir H, Bush AI, Conrad M, Dixon SJ, Fulda S, Gascon S, Hatzios SK, Kagan VE (2017). Ferroptosis: a regulated cell death Nexus linking metabolism, redox biology, and disease. Cell.

[112] Ning X, Zhang H, Wang C, Song X (2018). Exosomes released by gastric Cancer cells induce transition of Pericytes into Cancer-associated fibroblasts. Med Sci Monit.

[113] Shimoda A, Ueda K, Nishiumi S, Murata-Kamiya N, Mukai SA, Sawada S, Azuma T, Hatakeyama M, Akiyoshi K (2016). Exosomes as nanocarriers for systemic delivery of the helicobacter pylori virulence factor CagA. Sci Rep.

[114] Zheng P, Luo Q, Wang W, Li J, Wang T, Wang P, Chen L, Zhang P, Chen H, Liu Y (2018). Tumor-associated macrophages-derived exosomes promote the migration of gastric cancer cells by transfer of functional Apolipoprotein E. Cell Death Dis.

[115] Che Y, Geng B, Xu Y, Miao X, Chen L, Mu X, Pan J, Zhang C, Zhao T, Wang C (2018). Helicobacter pylori-induced exosomal MET educates tumour-associated macrophages to promote gastric cancer progression. J Cell Mol Med.

[116] Yen EY, Miaw SC, Yu JS, Lai IR (2017). Exosomal TGF-beta1 is correlated with lymphatic metastasis of gastric cancers. Am J Cancer Res.

[117] Gu J, Qian H, Shen L, Zhang X, Zhu W, Huang L, Yan Y, Mao F, Zhao C, Shi Y, Xu W (2012). Gastric cancer exosomes trigger differentiation of umbilical cord derived mesenchymal stem cells to carcinoma-associated fibroblasts through TGF-beta/Smad pathway. PLoS One.

[118] Wang JJ, Wang ZY, Chen R, Xiong J, Yao YL, Wu JH, Li GX (2015). Macrophage-secreted Exosomes delivering miRNA-21 inhibitor can regulate BGC-823 cell proliferation. Asian Pac J Cancer Prev.

[119] Liu F, Bu Z, Zhao F, Xiao D (2018). Increased T-helper 17 cell differentiation mediated by exosome-mediated microRNA-451 redistribution in gastric cancer infiltrated T cells. Cancer Sci.

[120] Wang J, Guan X, Zhang Y, Ge S, Zhang L, Li H, Wang X, Liu R, Ning T, Deng T (2018). Exosomal miR-27a derived from gastric Cancer cells regulates the transformation of fibroblasts into Cancer-associated fibroblasts. Cell Physiol Biochem.

[121] Zhu M, Zhang N, He S, Lu X (2020). Exosomal miR-106a derived from gastric cancer promotes peritoneal metastasis via direct regulation of Smad7. Cell Cycle.

[122] Shi L, Wang Z, Geng X, Zhang Y, Xue Z (2020). Exosomal miRNA-34 from cancer-associated fibroblasts inhibits growth and invasion of gastric cancer cells in vitro and in vivo. Aging (Albany NY).

[123] Xu G, Zhang B, Ye J, Cao S, Shi J, Zhao Y, Wang Y, Sang J, Yao Y, Guan W (2019). Exosomal miRNA-139 in cancer-associated fibroblasts inhibits gastric cancer progression by repressing MMP11 expression. Int J Biol Sci.

[124] Xu X, Cheng J, Luo S, Huang D, Xu J, Qian Y, Zhou H, Wan X (2020). Deoxycholic acid-stimulated macrophage-derived exosomes promote intestinal metaplasia and suppress pr oliferation in human gastric epithelial cells. Eur J Pharmacol.

[125] Li Z, Suo B, Long G, Gao Y, Song J, Zhang M, Feng B, Shang C, Wang D (2020). Exosomal miRNA-16-5p derived from M1 macrophages enhances T cell-dependent immune response by regulating PD-L1 in gastric Cancer. Front Cell Dev Biol.

[126] Zhang H, Zhu L, Bai M, Liu Y, Zhan Y, Deng T, Yang H, Sun W, Wang X, Zhu K (2019). Exosomal circRNA derived from gastric tumor promotes white adipose browning by targeting the miR-133/PRDM16 pathway. Int J Cancer.

[127] Ma S, McGuire MH, Mangala LS, Lee S, Stur E, Hu W, Bayraktar E, Villar-Prados A, Ivan C, Wu SY (2021). Gain-of-function p53 protein transferred via small extracellular vesicles promotes conversion of fibroblasts to a cancer-associated phenotype. Cell Rep.

[128] Hatakeyama M (2004). Oncogenic mechanisms of the helicobacter pylori CagA protein. Nat Rev Cancer.

[129] Li T, Guo H, Li H, Jiang Y, Zhuang K, Lei C, Wu J, Zhou H, Zhu R, Zhao X (2019). MicroRNA-92a-1-5p increases CDX2 by targeting FOXD1 in bile acids-induced gastric intestinal metaplasia. Gut.

[130] Chen C, Xu ZQ, Zong YP, Ou BC, Shen XH, Feng H, Zheng MH, Zhao JK, Lu AG (2019). CXCL5 induces tumor angiogenesis via enhancing the expression of FOXD1 mediated by the AKT/NF-kappaB pathway in colorectal cancer. Cell Death Dis.

[131] Hinata M, Kunita A, Abe H, Morishita Y, Sakuma K, Yamashita H, et al. Exosomes of Epstein-Barr virus-associated gastric carcinoma suppress dendritic cell maturation. Microorganisms. 2020;8:1776. 10.3390/microorganisms8111776 PMC7697542 33198173

[132] Liu J, Wu S, Zheng X, Zheng P, Fu Y, Wu C, Lu B, Ju J, Jiang J (2020). Immune suppressed tumor microenvironment by exosomes derived from gastric cancer cells via modulating immune functions. Sci Rep.

[133] Yang E, Wang X, Gong Z, Yu M, Wu H, Zhang D (2020). Exosome-mediated metabolic reprogramming: the emerging role in tumor microenvironment remodeling and its influence on cancer progression. Signal Transduct Target Ther.

[134] Xu Z, Zeng S, Gong Z, Yan Y (2020). Exosome-based immunotherapy: a promising approach for cancer treatment. Mol Cancer.

[135] Li X, Shao C, Shi Y, Han W (2018). Lessons learned from the blockade of immune checkpoints in cancer immunotherapy. J Hematol Oncol.

[136] Fan Y, Liu Y, Qu X (2019). ASO author reflections: the prognostic role of Exosomal PD-L1 in patients with gastric Cancer. Ann Surg Oncol.

[137] Fan Y, Che X, Qu J, Hou K, Wen T, Li Z, Li C, Wang S, Xu L, Liu Y, Qu X (2019). Exosomal PD-L1 retains immunosuppressive activity and is associated with gastric Cancer prognosis. Ann Surg Oncol.

[138] Zhang M, Fan Y, Che X, Hou K, Zhang C, Li C, Wen T, Wang S, Cheng Y, Liu Y, Qu X (2020). 5-FU-induced Upregulation of Exosomal PD-L1 causes immunosuppression in advanced gastric Cancer patients. Front Oncol.

[139] Yu W, Hurley J, Roberts D, Chakrabortty SK, Enderle D, Noerholm M, et al. Exosome-based liquid biopsies in cancer: opportunities and challenges. Ann Oncol. 2021;32:466–77. 10.1016/j.annonc.2021.01.074 PMC8268076 33548389

[140] Pan S, Zhang Y, Natalia A, Lim CZJ, Ho NRY, Chowbay B, et al. Extracellular vesicle drug occupancy enables real-time monitoring of targeted cancer therapy. Nat Nanotechnol. 2021. 10.1038/s41565-021-00872-w 33686255

[141] Zhou X, Zhu W, Li H, Wen W, Cheng W, Wang F, Wu Y, Qi L, Fan Y, Chen Y (2015). Diagnostic value of a plasma microRNA signature in gastric cancer: a microRNA expression analysis. Sci Rep.

[142] Dong Z, Sun X, Xu J, Han X, Xing Z, Wang D, Ge J, Meng L, Xu X (2019). Serum membrane type 1-matrix metalloproteinase (MT1-MMP) mRNA protected by Exosomes as a potential biomarker for gastric Cancer. Med Sci Monit.

[143] Huang Z, Zhu D, Wu L, He M, Zhou X, Zhang L, Zhang H, Wang W, Zhu J, Cheng W (2017). Six serum-based miRNAs as potential diagnostic biomarkers for gastric Cancer. Cancer Epidemiol Biomark Prev.

[144] Piao HY, Guo S, Wang Y, Zhang J (2020). Exosomal Long non-coding RNA CEBPA-AS1 inhibits tumor apoptosis and functions as a non-invasive biomarker for diagnosis of gastric Cancer. Onco Targets Ther.

[145] Zhao R, Zhang Y, Zhang X, Yang Y, Zheng X, Li X, Liu Y, Zhang Y (2018). Exosomal long noncoding RNA HOTTIP as potential novel diagnostic and prognostic biomarker test for gastric cancer. Mol Cancer.

[146] Li S, Zhang M, Zhang H, Hu K, Cai C, Wang J, Shi L, Ma P, Xu Y, Zheng P (2020). Exosomal long noncoding RNA lnc-GNAQ-6:1 may serve as a diagnostic marker for gastric cancer. Clin Chim Acta.

[147] Wang J, Zhang H, Zhou X, Wang T, Zhang J, Zhu W, Zhu H, Cheng W (2018). Five serum-based miRNAs were identified as potential diagnostic biomarkers in gastric cardia adenocarcinoma. Cancer Biomark.

[148] Li W, Gao YQ (2018). MiR-217 is involved in the carcinogenesis of gastric cancer by down-regulating CDH1 expression. Kaohsiung J Med Sci.

[149] Lu X, Lu J, Wang S, Zhang Y, Ding Y, Shen X, Jing R, Ju S, Chen H, Cong H (2021). Circulating serum exosomal miR-92a-3p as a novel biomarker for early diagnosis of gastric cancer. Future Oncol.

[150] Zheng GD, Xu ZY, Hu C, Lv H, Xie HX, Huang T, Zhang YQ, Chen GP, Fu YF, Cheng XD (2021). Exosomal miR-590-5p in serum as a biomarker for the diagnosis and prognosis of gastric Cancer. Front Mol Biosci.

[151] Cai C, Zhang H, Zhu Y, Zheng P, Xu Y, Sun J, Zhang M, Lan T, Gu B, Li S, Ma P (2019). Serum Exosomal Long noncoding RNA pcsk2-2:1 as a potential novel diagnostic biomarker for gastric Cancer. Onco Targets Ther.

[152] Wang N, Wang L, Yang Y, Gong L, Xiao B, Liu X (2017). A serum exosomal microRNA panel as a potential biomarker test for gastric cancer. Biochem Biophys Res Commun.

[153] Li Q, Shao Y, Zhang X, Zheng T, Miao M, Qin L, Wang B, Ye G, Xiao B, Guo J (2015). Plasma long noncoding RNA protected by exosomes as a potential stable biomarker for gastric cancer. Tumour Biol.

[154] Yamamoto H, Watanabe Y, Oikawa R, Morita R, Yoshida Y, Maehata T, Yasuda H, Itoh F (2016). BARHL2 methylation using gastric wash DNA or gastric juice Exosomal DNA is a useful marker for early detection of gastric Cancer in an H. pylori-independent manner. Clin Transl Gastroenterol.

[155] Guo X, Lv X, Ru Y, Zhou F, Wang N, Xi H, et al. Circulating Exosomal gastric Cancer-associated Long noncoding RNA1 as a biomarker for early detection and monitoring progression of gastric Cancer: a multiphase study. JAMA Surg. 2020;155:572–9. 10.1001/jamasurg.2020.1133 PMC7287948 32520332

[156] Tang S, Cheng J, Yao Y, Lou C, Wang L, Huang X, Zhang Y (2020). Combination of four serum Exosomal MiRNAs as novel diagnostic biomarkers for early-stage gastric Cancer. Front Genet.

[157] Shi Y, Wang Z, Zhu X, Chen L, Ma Y, Wang J, Yang X, Liu Z (2020). Exosomal miR-1246 in serum as a potential biomarker for early diagnosis of gastric cancer. Int J Clin Oncol.

[158] Shao Y, Tao X, Lu R, Zhang H, Ge J, Xiao B, Ye G, Guo J (2020). Hsa_circ_0065149 is an Indicator for early gastric Cancer screening and prognosis prediction. Pathol Oncol Res.

[159] Lin LY, Yang L, Zeng Q, Wang L, Chen ML, Zhao ZH, Ye GD, Luo QC, Lv PY, Guo QW (2018). Tumor-originated exosomal lncUEGC1 as a circulating biomarker for early-stage gastric cancer. Mol Cancer.

[160] Zhang Y, Han T, Feng D, Li J, Wu M, Peng X, Wang B, Zhan X, Fu P (2020). Screening of non-invasive miRNA biomarker candidates for metastasis of gastric cancer by small RNA sequencing of plasma exosomes. Carcinogenesis.

[161] Yun J, Han SB, Kim HJ, Go SI, Lee WS, Bae WK, Cho SH, Song EK, Lee OJ, Kim HK (2019). Exosomal miR-181b-5p Downregulation in ascites serves as a potential diagnostic biomarker for gastric Cancer-associated malignant ascites. J Gastric Cancer.

[162] Kumata Y, Iinuma H, Suzuki Y, Tsukahara D, Midorikawa H, Igarashi Y, Soeda N, Kiyokawa T, Horikawa M, Fukushima R (2018). Exosomeencapsulated microRNA23b as a minimally invasive liquid biomarker for the prediction of recurrence and prognosis of gastric cancer patients in each tumor stage. Oncol Rep.

[163] Tang W, Fu K, Sun H, Rong D, Wang H, Cao H (2018). CircRNA microarray profiling identifies a novel circulating biomarker for detection of gastric cancer. Mol Cancer.

[164] Zheng P, Zhang H, Gao H, Sun J, Li J, Zhang X, Gao L, Ma P, Li S (2020). Plasma Exosomal Long noncoding RNA lnc-SLC2A12-10:1 as a novel diagnostic biomarker for gastric Cancer. Onco Targets Ther.

[165] Xu H, Zhou J, Tang J, Min X, Yi T, Zhao J, Ren Y (2020). Identification of serum exosomal lncRNA MIAT as a novel diagnostic and prognostic biomarker for gastric cancer. J Clin Lab Anal.

[166] Tao X, Shao Y, Lu R, Ye Q, Xiao B, Ye G, Guo J (2020). Clinical significance of hsa_circ_0000419 in gastric cancer screening and prognosis estimation. Pathol Res Pract.

[167] Liu X, Chu KM (2020). Exosomal miRNAs as circulating biomarkers for prediction of development of haematogenous metastasis after surgery for stage II/III gastric cancer. J Cell Mol Med.

[168] Ohzawa H, Kumagai Y, Yamaguchi H, Miyato H, Sakuma Y, Horie H, Hosoya Y, Kawarai Lefor A, Sata N, Kitayama J (2020). Exosomal microRNA in peritoneal fluid as a biomarker of peritoneal metastases from gastric cancer. Ann Gastroenterol Surg.

[169] Ding XQ, Wang ZY, Xia D, Wang RX, Pan XR, Tong JH (2020). Proteomic profiling of serum Exosomes from patients with metastatic gastric Cancer. Front Oncol.

[170] Chanteloup G, Cordonnier M, Isambert N, Bertaut A, Hervieu A, Hennequin A, Luu M, Zanetta S, Coudert B, Bengrine L (2020). Monitoring HSP70 exosomes in cancer patients' follow up: a clinical prospective pilot study. J Extracell Vesicles.

[171] Keerthikumar S, Chisanga D, Ariyaratne D, Al Saffar H, Anand S, Zhao K, Samuel M, Pathan M, Jois M, Chilamkurti N (2016). ExoCarta: a web-based compendium of Exosomal cargo. J Mol Biol.

[172] Blanco E, Shen H, Ferrari M (2015). Principles of nanoparticle design for overcoming biological barriers to drug delivery. Nat Biotechnol.

[173] Guan XW, Zhao F, Wang JY, Wang HY, Ge SH, Wang X, Zhang L, Liu R, Ba Y, Li HL (2017). Tumor microenvironment interruption: a novel anti-cancer mechanism of proton-pump inhibitor in gastric cancer by suppressing the release of microRNA-carrying exosomes. Am J Cancer Res.

[174] Qiao L, Hu S, Huang K, Su T, Li Z, Vandergriff A, Cores J, Dinh PU, Allen T, Shen D (2020). Tumor cell-derived exosomes home to their cells of origin and can be used as Trojan horses to deliver cancer drugs. Theranostics.

[175] Geng T, Pan P, Leung E, Chen Q, Chamley L, Wu Z (2021). Recent advancement and technical challenges in developing small extracellular vesicles for Cancer drug delivery. Pharm Res.

[176] Cabeza L, Perazzoli G, Pena M, Cepero A, Luque C, Melguizo C, Prados J (2020). Cancer therapy based on extracellular vesicles as drug delivery vehicles. J Control Release.

[177] Dai J, Su Y, Zhong S, Cong L, Liu B, Yang J, Tao Y, He Z, Chen C, Jiang Y (2020). Exosomes: key players in cancer and potential therapeutic strategy. Signal Transduct Target Ther.

[178] Shehzad A, Islam SU, Shahzad R, Khan S, Lee YS (2021). Extracellular vesicles in cancer diagnostics and therapeutics. Pharmacol Ther.

[179] Chen W, Jiang J, Xia W, Huang J (2017). Tumor-related Exosomes contribute to tumor-promoting microenvironment: an immunological perspective. J Immunol Res.

[180] Whiteside TL (2016). Exosomes and tumor-mediated immune suppression. J Clin Invest.

[181] Olejarz W, Dominiak A, Zolnierzak A, Kubiak-Tomaszewska G, Lorenc T (2020). Tumor-derived Exosomes in immunosuppression and immunotherapy. J Immunol Res.

[182] Andre F, Chaput N, Schartz NE, Flament C, Aubert N, Bernard J, Lemonnier F, Raposo G, Escudier B, Hsu DH (2004). Exosomes as potent cell-free peptide-based vaccine. I. Dendritic cell-derived exosomes transfer functional MHC class I/peptide complexes to dendritic cells. J Immunol.

[183] Cheng L, Wang Y, Huang L (2017). Exosomes from M1-polarized macrophages potentiate the Cancer vaccine by creating a pro-inflammatory microenvironment in the lymph node. Mol Ther.

[184] Wiklander OP, Nordin JZ, O'Loughlin A, Gustafsson Y, Corso G, Mager I, Vader P, Lee Y, Sork H, Seow Y (2015). Extracellular vesicle in vivo biodistribution is determined by cell source, route of administration and targeting. J Extracell Vesicles.

[185] Jayasinghe MK, Tan M, Peng B, Yang Y, Sethi G, Pirisinu M, et al. New approaches in extracellular vesicle engineering for improving the efficacy of anti-cancer therapies. Semin Cancer Biol. 2021. 10.1016/j.semcancer.2021.02.010 33609665

[186] Liang Y, Duan L, Lu J, Xia J (2021). Engineering exosomes for targeted drug delivery. Theranostics.

[187] Zhang X, Zhang H, Gu J, Zhang J, Shi H, Qian H, et al. Engineered extracellular vesicles for Cancer therapy. Adv Mater. 2021;33:e2005709. 10.1002/adma.202005709 33644908

[188] Elsharkasy OM, Nordin JZ, Hagey DW, de Jong OG, Schiffelers RM, Andaloussi SE, Vader P (2020). Extracellular vesicles as drug delivery systems: why and how?. Adv Drug Deliv Rev.

[189] Pham TC, Jayasinghe MK, Pham TT, Yang Y, Wei L, Usman WM, Chen H, Pirisinu M, Gong J, Kim S (2021). Covalent conjugation of extracellular vesicles with peptides and nanobodies for targeted therapeutic delivery. J Extracell Vesicles.

[190] Wang J, Li W, Zhang L, Ban L, Chen P, Du W, Feng X, Liu BF (2017). Chemically edited Exosomes with dual ligand purified by microfluidic device for active targeted drug delivery to tumor cells. ACS Appl Mater Interfaces.

[191] Nasiri Kenari A, Cheng L, Hill AF (2020). Methods for loading therapeutics into extracellular vesicles and generating extracellular vesicles mimetic-nanovesicles. Methods.

[192] Pan S, Pei L, Zhang A, Zhang Y, Zhang C, Huang M, Huang Z, Liu B, Wang L, Ma L (2020). Passion fruit-like exosome-PMA/au-BSA@Ce6 nanovehicles for real-time fluorescence imaging and enhanced targeted photodynamic therapy with deep penetration and superior retention behavior in tumor. Biomaterials.

[193] Kim S, Sohn HJ, Lee HJ, Sohn DH, Hyun SJ, Cho HI, Kim TG (2017). Use of engineered Exosomes expressing HLA and Costimulatory molecules to generate antigen-specific CD8+ T cells for adoptive cell therapy. J Immunother.

[194] Nguyen VVT, Witwer KW, Verhaar MC, Strunk D, van Balkom BWM (2020). Functional assays to assess the therapeutic potential of extracellular vesicles. J Extracell Vesicles.

